# p190-B RhoGAP and intracellular cytokine signals balance hematopoietic stem and progenitor cell self-renewal and differentiation

**DOI:** 10.1038/ncomms14382

**Published:** 2017-02-08

**Authors:** Ashwini Hinge, Juying Xu, Jose Javier, Eucabeth Mose, Sachin Kumar, Reuben Kapur, Edward F. Srour, Punam Malik, Bruce J. Aronow, Marie-Dominique Filippi

**Affiliations:** 1 Division of Experimental Hematology and Cancer Biology, Cincinnati Children's Research Foundation, University of Cincinnati College of Medicine, Cincinnati, Ohio 45229, USA; 2 Department of Medicine, Indiana University School of Medicine, Indianapolis, Indiana 46202, USA; 3 Division of Biomedical Informatics, Cincinnati Children's Research Foundation, University of Cincinnati College of Medicine, Cincinnati, Ohio 45229, USA

## Abstract

The mechanisms regulating hematopoietic stem and progenitor cell (HSPC) fate choices remain ill-defined. Here, we show that a signalling network of p190-B RhoGAP-ROS-TGF-β-p38^MAPK^ balances HSPC self-renewal and differentiation. Upon transplantation, HSPCs express high amounts of bioactive TGF-β1 protein, which is associated with high levels of p38^MAPK^ activity and loss of HSC self-renewal *in vivo*. Elevated levels of bioactive TGF-β1 are associated with asymmetric fate choice *in vitro* in single HSPCs via p38MAPK activity and this is correlated with the asymmetric distribution of activated p38MAPK. In contrast, loss of p190-B, a RhoGTPase inhibitor, normalizes TGF-β levels and p38MAPK activity in HSPCs and is correlated with increased HSC self-renewal *in vivo*. Loss of p190-B also promotes symmetric retention of multi-lineage capacity in single HSPC myeloid cell cultures, further suggesting a link between p190-B-RhoGAP and non-canonical TGF-β signalling in HSPC differentiation. Thus, intracellular cytokine signalling may serve as ‘fate determinants' used by HSPCs to modulate their activity.

## Abstract (web summary)

The success of hematopoietic stem cell (HSC) transplantation relies on understanding what regulates the fate decision to self-renew. Here, the authors show using both *in vitro* assays and *in vivo* transplantation that loss of the RhoGAP p190-B enhances self-renewal by inhibiting TGFβ/p38 signalling.

Hematopoietic stem cells (HSCs) are multipotent cells that provide life-long blood and immune cells by their ability to regenerate themselves—that is, self-renew—and to differentiate into a variety of mature cells^1^. This potential has allowed the development of clinical HSC transplantation, the success of which depends on HSC numbers and their self-renewing activity^2^,^3^. Therefore, understanding the mechanisms that control HSC self-renewal is of particular biological and clinical importance.

Although HSCs are mostly in a quiescent state^4^, HSC fate decisions to self-renew or to commit to differentiation happen during cell division. These fate decisions must be tightly regulated to regenerate the pool of HSCs and produce adequate numbers of mature blood cells at steady state or during stress-induced regeneration^1^,^5^. HSC quiescence, survival and self-renewal are controlled by separate pathways, although they are integrated^6^,^7^. Understanding how the balance between HSC self-renewal and differentiation is regulated remains a central issue in HSC biology. Asymmetric self-renewal division enables HSCs to produce distinct daughter cells, one that will maintain the features of a HSC and one that will commit to differentiation, through unequal inheritance of fate determinants by daughter cells. It is thought that HSCs may modulate their fate and generate either two stem cells or two committed progenitors to meet the demand. Recently, numerous studies suggest the occurrence of both symmetric self-renewal division and asymmetric self-renewal division *in vitro* and *in vivo*^6^,^8^,^9^,^10^.

A challenge in investigating HSC fate choices has been that HSCs are retrospectively defined by their ability to generate all mature cells, making assessments of HSC state highly dependent on the proliferation and differentiation potential of the immediate progeny^6^. Further, accumulating evidence points to the heterogeneity of the HSC compartment^11^,^12^,^13^,^14^. Thus, identification of networks that regulate HSC fate decisions requires HSC analysis under conditions where progenitor proliferation and differentiation are unchanged. Studies at the single cell level have provided valuable information on HSC self-renewal, revealing stem cell factor (SCF) signalling intensity, Lnk signalling pathway and lipid metabolism are important for HSC fate^6^,^8^,^9^,^15^,^16^. However, only few studies have identified factors that alter HSC fate and are asymmetrically segregated at cell division. Recently, occurrence of asymmetric segregation of the endocytic marker Ap2a2 associated with changes in HSC fate has been reported^17^.

Members of the Rho GTPase family are critical regulators of HSC functions^18^,^19^,^20^,^21^. They cycle between an active GTP-bound and an inactive GDP-bound state^22^. Guanine nucleotide exchange factors (GEFs) promote the exchange of GDP for GTP whereas GTPase-activating proteins (GAPs) accelerate the rate of hydrolysis of GTP. Rho GTPases are best known for their roles in cytoskeleton reorganization, and contribute to the regulation of asymmetric cell division^23^. Our laboratory previously reported that p190-B RhoGAP (p190-B), a negative regulator of Rho GTPase signalling^24^,^25^, limits HSC self-renewal^19^. Interestingly, loss of p190-B enhanced long-term engraftment without altering HSC quiescence, proliferation, survival and their mature lineage differentiation potential^19^, making it an ideal model to study HSPC functions that are inherited through divisions.

Here, an *in vitro* assay of paired daughter cells at the clonal level coupled with *in vivo* transplantation and gene profiling experiments were used to identify regulatory networks of hematopoietic stem and progenitor cell (HSPC) activity during bone marrow (BM) regeneration.

We identified a novel mechanism of HSPC regulation, where TGF-β proteins are produced by HSPC *in vivo*, downstream of p190-B and reactive oxygen species (ROS), during BM regeneration, and signal through non-canonical p38^MAPK^ pathway to alter HSPC functions independent on cell cycle *in vivo*, and modulate retention of multiple myeloid lineages in single HSPC *in vitro*. Intriguingly, this is correlated with occurrence of asymmetric segregation of p38^MAPK^ activity to daughter cells during HSPC divisions *in vitro*. This study implies that HSCs produce stress cytokines to autonomously modulate signalling pathways during HSC regeneration, and reveals novel functions for non-canonical TGF-β signalling as ‘fate determinant' of HSPC functions uncoupled from HSPC quiescence.

## Results

### p190-B regulates HSPC activity independent of proliferation

**Figure 1:**
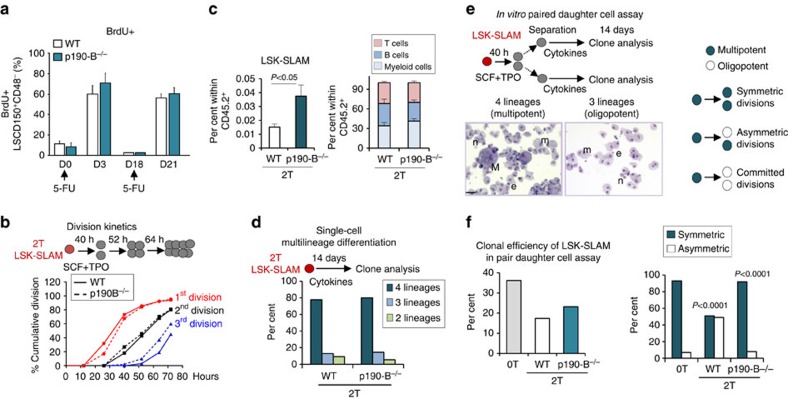
p190-B regulates HSC self-renewal independent of proliferation. WT and p190-B^−/−^ fetal liver cells were used for serial competitive transplantation as in ref. 19. (at least two independent experiments) (**a**) BrdU incorporation was performed in secondary transplanted (2T) WT and p190-B^−/−^ mice challenged with 5-FU at indicated timeto examine HSPC proliferation. BM was harvested 18 h after BrdU treatment at each time point, stained and analysed by flow cytometry (mean±s.e.m.; *n*=5 mice per group). (**b**) Cell division kinetics. Single LSK-SLAM cells from 2T-WT and 2T- p190B^−/−^ transplanted mice were isolated. Cells were counted every 12 h to determine division kinetics (*n*=75–100 cells per group from three independent experiments). (**c**) Frequency of LSK-SLAM and per cent lineage reconstitution in BM of 2T mice with WT and p190-B^−/−^ cells, 4 months post-transplant. (mean±s.e.m.; *n*=7 mice per group). (**d**) Single cell multilineage differentiation assay. Single LSK-SLAM cells were isolated and cultured with serum and multiple cytokines to induce terminal myeloid differentiation, for 14 days. Bar graphs show per cent of clones containing 4, 3 and 2 lineages initiated from LSK-SLAM cells (*n*=50–60 clones per group). (**e**,**f**) *In vitro* paired daughter cell assay of single LSK-SLAM cells isolated from control (0T, non-transplanted cells) and 2T-WT and 2T- p190-B^−/−^ mice. Paired-daughter cells were separated and further cultured individually with serum and multiple cytokines to induce terminal myeloid differentiation, for 14 days. (**e**) Schema of the assay; images illustrate an asymmetric division with one multi-potent clone containing four myeloid lineages (e: erythroid cells, n: neutrophils, m: macrophage/monocyte, M: megakaryocyte), and the daughter clone containing only three lineages (n,e,m), scale bar, 20 μm. (**f**) Left bar graph shows per cent of cloning efficiency of single cells generating paired-daughter clones; bar graph on the right shows relative frequency of asymmetric and symmetric progenitor divisions calculated from cells generating at least one multipotent daughter cell (*n*=35–55 pairs per group from three or more independent experiments). *P* values were calculated by Fisher exact 2 × 2 contingency table by comparing percent of symmetric and asymmetric divisions of the following groups: 2T-WT versus 0T and 2T-p190-B^−/−^ versus 2T-WT.

We used a combination of *in vitro* single cell culture assays and *in vivo* long-term repopulation experiments to investigate the role of signalling pathways on HSPC functions. HSC self-renewal is functionally identified in the serial repopulation assay, which tests the capacity of HSCs to provide life-long reconstitution of all blood-cell lineages and to maintain these properties in secondary recipients. Since HSC self-renewal capacity is finite, a decline in HSC activity is generally observed over serial competitive repopulation assay. We previously reported that p190-B loss enhances HSC self-renewal during serial transplantation^19^. These experiments were performed with fetal liver hematopoietic cells as p190-B-deficiency is embryonic lethal^24^,^25^. However, this phenotype is not restricted to fetal liver HSPCs since LSK (Lineage^−^Sca-1^+^c-Kit^+^) from p190-B haploinsufficient adult animals gave rise to higher long-term engraftment than LSK from wild-type (WT) mice (Supplementary Fig. 1A). A classical cause of HSC exhaustion is proliferative stress or inability to return to quiescence following hematopoietic regeneration^26^. However, p190-B-deficiency does not alter phenotypically defined HSPCs (LSK-CD150^+^CD48^–^ [LSK-SLAM]) survival and proliferation *in vitro* and *in vivo*^19^. Here, to further evaluate this, mice transplanted with WT or p190-B^−/−^ cells were treated with the myeloablative 5-fluorouracile (5FU) to induce LSK-SLAM proliferation. Three days following 5FU challenge, WT and p190-B^−/−^ LSK-SLAM incorporated BrdU at the same level. Eighteen days later, LSK-SLAM from each group had returned to quiescence. A second 5FU treatment induced similar WT and p190-B^−/−^ LSK-SLAM proliferation (Fig. 1a). *In vitro* on the single cell level, the kinetics of the first division of 2T-LSK-SLAM isolated from secondary transplanted animals (2T) was identical between the genotypes (Fig. 1b). Yet, p190-B deletion prevented LSK-SLAM depletion and maintained normal proportion of blood lineages over transplantation (Fig. 1c). Hence, p190-B controls HSC self-renewal independent of HSC quiescence and proliferation, making it an ideal model to examine mechanisms of HSPC functions during divisions.

HSC fate decisions to commit to differentiation—or not—occur during division^5^,^27^. To investigate this, we examined lineage differentiation potential of LSK-SLAM and of their immediate progeny at the clonal level using *in vitro* assays described by Drs Suda and Nakauchi^9^,^15^,^28^. In one set of experiments, single LSK-SLAM cells were cultured with multiple cytokines (SCF, TPO, IL-3, G-CSF, EPO) and serum to promote their proliferation and differentiation toward myeloid cell lineages, for 14 days. Under these conditions, single cells generated clones that contain erythroid cells (e), neutrophils (n), macrophages (m) and megakaryocytes (M). In another set of experiments, single LSK-SLAM cells were first cultured in serum-free medium with SCF and TPO for the time of one division; the daughter cells were then separated into two wells and further cultured with SCF, TPO, IL-3, G-CSF, EPO and serum to determine lineage differentiation potential of each daughter cell, known as ‘*in vitro* paired daughter cell assay'. Under these conditions, single LSK-SLAM can divide symmetrically and produce two daughter cells that have multiple myeloid lineage potential (hereafter nemM daughter cell)^9^. Alternatively, cells can divide asymmetrically and generate one nemM daughter cell and one daughter cell that is committed to specific lineage differentiation. Some cells also generate two committed daughter cells. This *in vitro* assay measures symmetric or asymmetric retention of multiple myeloid lineages in single HSPC. This assay does not measure, nor can it be used to infer, any impact on self-renewal of LT-HSC. This assay does not account for the role of the microenvironment. Nevertheless, previous work from the Eaves and Nakauchi groups have shown on the single cell level that HSCs can be retained *in vitro* with similar ‘supra-physiological' levels of cytokines^6^,^10^,^11^,^15^. Since these analyses depend on the formation of a colony *in vitro* in non-hypoxic conditions, which may cause bias in estimation of cell differentiation potential^9^, *in vitro* paired daughter cell assays of WT and p190-B-deficient cells were always assayed in parallel under exactly the same conditions. We first examined nemM potential of single 2T-LSK-SLAM cells. 2T-LSK-SLAM from each genotype had similar clonogenic efficiency, and these cells produced similar frequency of nemM clones (80%) (Fig. 1d), indicating that these cells are multipotent and that their descendant cells have comparable proliferation and differentiation potential, in response to serum and cytokines *in vitro*. We then analysed the multilineage potential of daughter cells of single LSK-SLAM division, in the *in vitro* paired daughter cell assay (Fig. 1e)^9^ relative to non-previously transplanted LSK-SLAM isolated from 6-week old mice (0T). The numbers of LSK-SLAM divisions that did not generate any nemM daughter cells were similar between the groups (∼10%, Supplementary Table 1). The cloning efficiency was similar between 2T-WT and 2T-p190-B^−/−^ LSK-SLAM (Fig. 1f). Within divisions generating at least one nemM daughter cell, 0T LSK-SLAM mostly generated two nemM daughter cells (93% symmetric divisions). 2T-WT LSK-SLAM produced only 51% symmetric divisions. 2T-p190-B^−/−^ LSK-SLAM maintained up to 92% symmetric divisions (Fig. 1f). Similar results were obtained with LSK-SLAM isolated from fetal livers or primary transplanted mice (Supplementary Fig. 1B–D). Hence, p190-B deficiency enhances multilineage potential inheritance during LSK-SLAM division *in vitro*. This cannot be explained by alteration in first cell division rate or differences in phenotype of descendant cells in response to serum and cytokines, suggesting that p190-B loss prevents more rapid LSK-SLAM differentiation *in vitro*.

### p190-B controls TGF-β signalling following HSC engraftment

**Figure 2:**
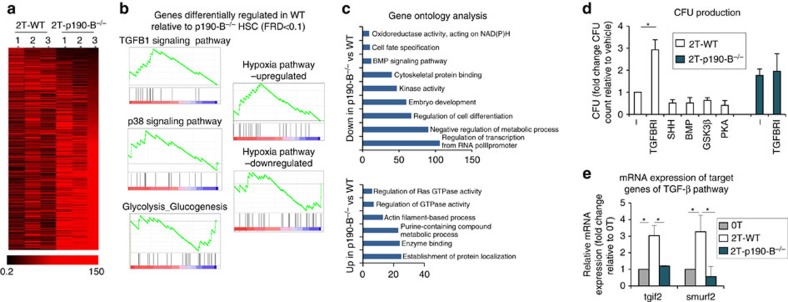
p190-B regulates TGF-β signaling following serial transplantation. (**a**–**c**) LSK-SLAM cells were isolated from three independent 2T mice per group, three independent times, and used for microarray analyses. (**a**) Heat map of genes differentially expressed, top candidate genes based on Student's *t*-test values. (**b**) Unbiased gene set enrichment analysis of differentially expressed gene, FDR<0.1. (**c**) Bar graphs show the gene ontology results for molecular and biological processes with the indicated numbers of genes that were different in 2T-p190-B^−/−^ HSC, of top differentially expressed candidate genes, analysed in TopGene Suite, *P*<0.0001. (**d**) CFU after *in vitro* culture. LSK-SLAM cells from 2T-WT mice were cultured with SCF+TPO for 4 days in the presence of inhibitors of various signaling pathways and cells from 2T-p190-B^−/−^ were treated with TGFBRI inhibitor 1;, and then plated in CFU assay without inhibitors to assess progenitor production. Data represented as fold change in CFUs of cultured cells compared with non-treated 2T-WT cells from 3 independent experiments (mean±s.e.m.). *P<0.05, two-tailed unpaired *t*-test. (**e**) mRNA expression analyses by qPCR of TGF-β signaling target genes—tgif2 and smurf2 in LSK cells isolated from control, 2T-WT and 2T-p190B^−/−^ mice. Data are normalized to β actin and presented as fold changes relative to non-transplanted cells. (mean±s.e.m.; *n*=3–5 from three independent experiments). *P<0.05, two-tailed unpaired *t*-test.

To identify how p190-B controls HSPC functions, we compared the transcription profile of 2T-WT and 2T-p190-B^−/−^ LSK-SLAM (Fig. 2a, Supplementary Data 1,2). As expected, genes categorizing ‘Rho GTPase signalling, cytoskeleton rearrangement' were differentially expressed between the genotypes. Unbiased gene set enrichment analysis (Fig. 2b) revealed that expression of gene sets associated to TGF-β and p38^MAPK^ signalling pathways were elevated in 2T-WT. Gene ontology analysis of top differentially expressed genes indicated that genes classified under ‘regulation of cell differentiation' were downregulated in 2T-p190-B^−/−^ LSK-SLAM (Fig. 2c). Interestingly, signalling pathways, including PKA, BMP, HHG and TGF-β pathways^3^,^29^, were also downregulated in 2T-p190-B^−/−^ LSK-SLAM. To test whether inhibiting these pathways restored the loss of WT transplanted HSPC activity, LSK-SLAM cells were cultured for 4 days with or without a series of pharmacological inhibitors. Hematopoietic potential of resulting cultures was tested in colony forming unit (CFU) assay. In this assay, 2T-p190-B^−/−^ LSK-SLAM produced two-fold more CFU than 2T-WT cells (Fig. 2d), as previously shown^19^. Surprisingly, inhibition of TGF-β signalling significantly increased CFU production of 2T-WT-LSK-SLAM cultures, but not that of 2T-p190-B^−/−^ LSK-SLAM cultures (Fig. 2d). Inhibition of other pathways had no effects. Consistently, 2T-WT LSK-SLAM expressed higher amount of mRNA of target genes of the TGF-β pathway—tgif2 and smurf2 (ref. 30)—than 2T-p190-B^−/−^ LSK and 0T LSK (Fig. 2e). mRNA expressions of TGF-β1, TGF-βRI, II and III, and expression of TGF-β RI (TGFBRI) at cell surface remained unchanged (Supplementary Fig. 2A,B). These data suggest a possible link between p190-B and TGF-β signalling in HSPC activity.

### p190-B controls HSPC activity via TGF-β signalling

**Figure 3:**
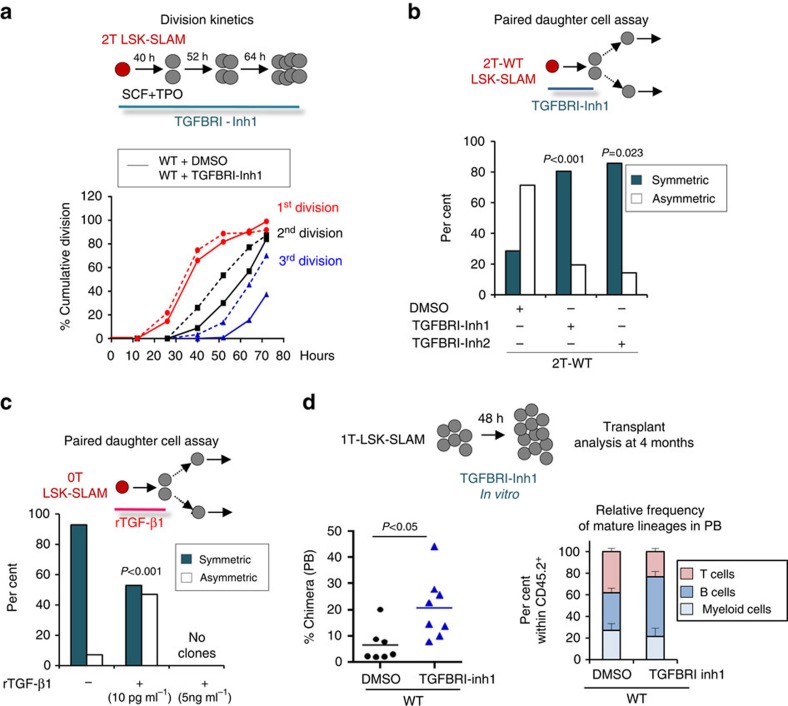
Loss of p190-B modulates HSPC activity via TGF-β signaling. (**a**) Effect of TGFBRI inhibitor 1 on cell division kinetics of LSK-SLAM isolated from 2T WT mice. Single cells were treated with TGFBRI inhibitor 1 or DMSO *ex vivo* for 72 h to determine division kinetics as in Fig. 1 (*n*=100 cells per group in 2 independent experiments). (**b**) Effect of TGFBRI inhibitor 1 on cell division output using the *in vitro* paired daughter cell assay. Single LSK-SLAM cells isolated from 2T-WT mice were treated with TGFBRI inhibitor 1 (*n*=36 pairs) or TGFBRI inhibitor 2 (*n*=7 pairs) or DMSO (*n*=14 pairs) for the duration of one division. Daughter cells were separated and further cultured individually with serum and cytokines without inhibitors to assess multilineage differentiation potential of daughter cells. (**c**) Effect of rTGF-β1 on 0T-LSK-SLAM division output using the paired daughter cell assay as in **b**. Single LSK-SLAM cells isolated from 0T-WT mice were treated with rTGF-β1 (10 pg ml^−1^ and 5 ng ml^−1^ *n*=18 pairs) for one division; daughter cells were analysed as in **b**. Bar graphs in B&C show per cent of asymmetric and symmetric divisions in each group, from at least two independent experiments. *P* values were calculated by Fisher exact 2 × 2 contingency table by comparing per cent of symmetric and asymmetric divisions of each inhibitor relative to DMSO in **b**, and rTGF-β1 treatment relative to control in **c**. (**d**) Schema of experimental design. LSK-SLAM were isolated from 1T-WT mice, cultured with SCF+TPO with TGFBRI inhibitor 1 (10 μM) or DMSO for 48 h and transplanted into recipients with competitor cells without inhibitor. Dot plot shows PB analysis 4 months post-transplant; per cent donor-cell chimera is shown. Bar graph shows donor-cell derived relative lineage reconstitution in PB, 4 months post-transplant (mean±s.e.m.; *n*=8 in 2 independent experiments). *P* value was calculated using 2-tailed unpaired *t* test.

Since genetic deletion of TGF-β signalling in hematopoietic cells induces a rapid and lethal inflammatory disorder, precluding long-term analysis of hematopoietic activity^31^, we used pharmacological inhibitors to assess the functional importance of TGF-β signalling on HSPC functions. This approach allowed us to transiently inhibit TGF-β signalling during LSK-SLAM division but not during the growth and differentiation of each paired-daughter cell after separation, in the *in vitro* paired-daughter cell assay. Inhibition of TGF-β signalling using the canonical inhibitor of TGFBRI kinase, SB431542^R^ [TGFBRI-Inh1] did not change the kinetics of the first division of 2T-WT LSK-SLAM, although it accelerated the third division rate (Fig. 3a). SB431542 completely rescued symmetric retention of multiple myeloid potential of single 2T-WT LSK-SLAM divisions *in vitro* (Fig. 3b). Similar results were obtained using another TGF-β RI kinase inhibitor II^R^ (TGFBRI-Inh2; Fig. 3b)^32^.

In contrast, recombinant TGF-β1 (rTGF-β1) shifted 0T LSK-SLAM divisions towards asymmetric divisions (Fig. 3c). This effect was seen at low concentrations of rTGF-β1 (10 pg ml^−1^) that do not induce LSK-SLAM hibernation. Otherwise, 5 ng ml^−1^ of rTGF-β1 did inhibit HSPC cell cycle. Wnt3a or Wnt5, important regulators of HSC functions, had no effect on 0T LSK-SLAM division outcome *in vitro*^33^,^34^. GSK3b inhibitor treatment did not change 2T-WT LSK-SLAM asymmetric divisions (Supplementary Fig. 3A,B). Hence, TGF-β1 signalling seems to play a specific role on nemM potential inheritance of LSK-SLAM divisions *in vitro*, which cannot be explained by alteration in first cell division kinetics.

To investigate the importance of this pathway on HSC activity, WT LSK-SLAM isolated from primary recipients were treated with SB431542 *ex vivo* during their first division only, and transplanted into secondary mice with competitor cells (Fig. 3d). As noted previously, this treatment did not change the kinetics of the initial LSK-SLAM division (Fig. 3a). Yet, TGFBRI inhibitor-treated cells gave rise to higher PB chimerism than cells treated with DMSO (vehicle control) at 16 weeks following engraftment (Fig. 3d). No differences in the relative proportion of blood lineages were noted (Fig. 3d). Thus, TGF-β inhibition maintained HSC activity through LSK-SLAM divisions *in vitro*, in pooled cultured cells.

### TGF-β RI inhibition promotes HSC regeneration *in vivo*

**Figure 4:**
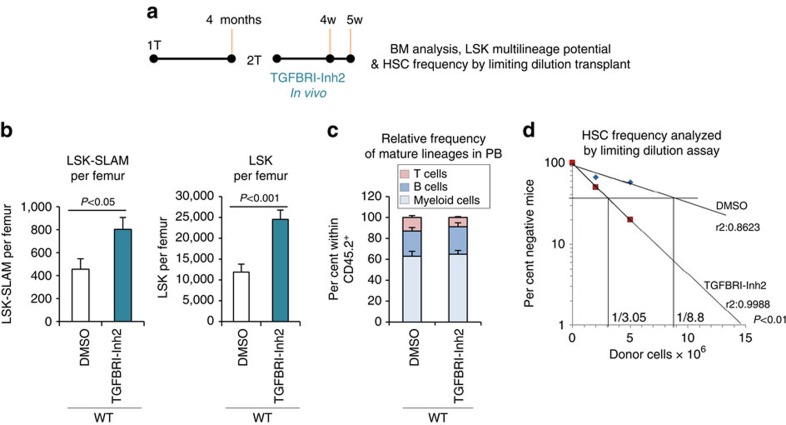
Inhibition of TGF-β signaling *in vivo* reverses loss of HSC self-renewal. Secondary recipient mice of WT cells were treated *in vivo* with DMSO or TGF-β RI kinase inhibitor II [TGFBRI-Inh2] for four weeks, BM was analysed one week later, data from 2–3 independent experiments. (**a**) Schema of experimental design. (**b**) LSK-SLAM and LSK numbers per femur in BM of 2T-WT mice treated with either DMSO or TGFBRI-Inh2 *in vivo*, five weeks post-transplant. (mean±s.e.m.; *n*=8–9 mice from 2 independent experiments). (**c**) Donor-cell derived relative lineage reconstitution in PB, 4 months post-transplant (mean±s.e.m.; *n*=8–9 mice from 2 independent experiments). (**d**) HSC frequency analysed by limiting competitive repopulation assay. BM from 2T-WT mice treated with TGFBRI-Inh2 or DMSO vehicle was used for tertiary transplant in competitive limiting dilution settings (x axis is in million donor cells per recipients, *n*=4–5 per group, per cell dose). Graph indicates percent negative mice in each group at different cell doses. (*n*=2 experiments, *P* value calculated by chi square by comparing frequency of negative mice between groups).

TGF-β signalling is important for HSC quiescence *in vivo*^35^, and for them to return to quiescence after chemotherapy, such that inhibiting TGFβ signalling after stress enhanced HSC pool and hematopoietic recovery^36^,^37^. Here, to determine whether TGFBRI inhibition can reverse loss of HSC self-renewal *in vivo*, TGFBRI-Inh2 (ref. 32) was injected into secondary transplanted mice during the time of HSC pool regeneration—4 weeks, and analysed 1 or 2 weeks later (Fig. 4a). TGFBRI-Inh2 treatment increased LSK-SLAM and LSK frequencies in BM (Fig. 4b) without affecting blood profile and BM cellularity (Supplementary Table 2, Supplementary Fig. 4A) or blood lineage content (Fig. 4c) or cell cycle of LSK-SLAM (Supplementary Fig. 4B). Importantly, it increased BM HSC frequency, as assessed by limiting dilution competitive repopulation experiments in tertiary recipients. Under these conditions, HSC activity is defined if the transplanted cells contribute to 1% or more of both lymphoid and myeloid lineages in the peripheral blood (PB)^11^,^38^. Donor BM cells from TGFBRI-Inh2 treated mice repopulated recipients at higher frequency than DMSO-treated group (Fig. 4d). These results suggest that TGFBRI inhibition conferred higher probability of HSC self-renewal *in vivo* (Supplementary Fig. 5).

### P190-B controls HSPC shape asymmetry via TGF-β signalling

Asymmetric cell divisions need polarized structures^39^,^40^. We thus examined cell shape, polymerized filamentous actin (F-actin) and microtubule organization in our model. Non-transplanted and 2T-p190-B^−/−^ LSK-SLAM (78–80%) appeared round with symmetrical cell shape. Interestingly, 2T-WT LSK-SLAM exhibited a more elongated shape with asymmetric distribution of F-actin (Supplementary Fig. 6A,B). TGFBR inhibitor treatment restored 2T-WT LSK-SLAM cell shape into a round structure (Supplementary Fig. 6C). Conversely, treatment with rTGF-β1 caused 0T LSK-SLAM to elongate (Supplementary Fig. 6D). Hence, cytoskeleton changes may be an important mechanism by which p190-B and TGF-β signalling regulate HSPC activity.

### p190-B deficiency prevents production of aTGF-β in HSPCs

**Figure 5:**
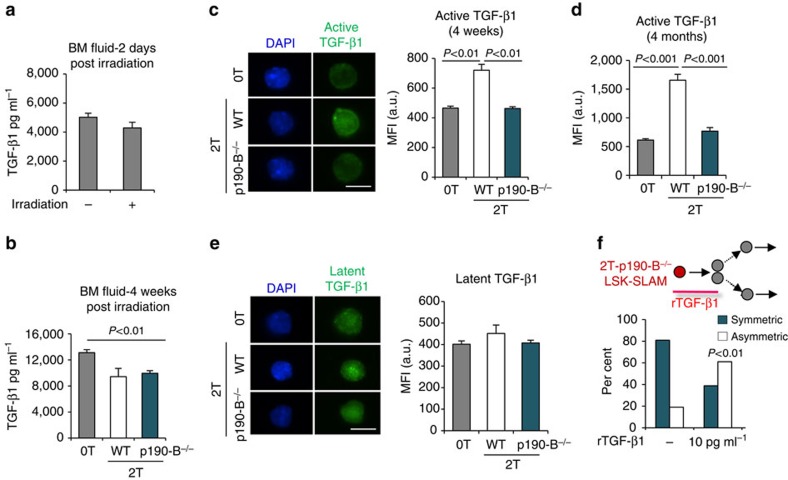
p190-B- deficiency prevents elevation of bioactive TGF-β in HSPCs. (**a**,**b**) aTGF-β1 levels were measured by ELISA in BM fluid of control (0T) mice, and in recipient mice of WT cells 2 days following irradiation and transplantation (**a**) or in secondary recipient mice of WT and p190-B^−/−^ cells 4 weeks following transplantation and in age matched control mice (**b**). Data are mean±s.e.m.; *n*=3 independent samples in each experiment, two-tailed unpaired *t*-test. (**c**,**d**) Detection of aTGF-β1 levels by immunofluorescence in LSK-SLAM 4 weeks (**c**) and 4 months (**d**) following transplantation. Representative images of LSK-SLAM isolated from control, 2T-WT and 2T-p190-B^−/−^ mice (bioactive TGF-β1 (green) and DAPI (blue), scale bar 10 μm). Bar graph shows quantification of mean fluorescence intensity in each group (C&D are mean±s.e.m. from *n*=2 independent experiments, 35–50 cells from each experiment, two-tailed unpaired *t*-test). (**e**) Detection of latent TGF-β1 in LSK-SLAM 4 months following transplantation, by immunofluorescence (latent TGF-β1 (green) and DAPI (blue). Bar graph shows quantification of mean fluorescence intensity in each group (mean±s.e.m.; 35–50 cells from each experiment were analysed; *n*=2 experiments). (**f**) Effect of rTGF-β on 2T-p190-B^−/−^ LSK-SLAM division output using the *in vitro* paired daughter cell assay as in Fig. 1. Single LSK-SLAM cells isolated from 2T-p190-B^−/−^ recipients were treated with rTGF-β1 (10 pg ml^−1^) for one division; daughter cells were analysed as in Fig. 1. (*n*=17 pairs from two independent experiments, fisher exact 2 × 2 contingency table).

TGF-β factors are secreted as inactive protein complex bound to the latency-associated peptide and the latent TGF-β1 binding protein-1 (LTBP1)^41^. Dissociation from the complex enables protein binding to their receptors, making a so-called ‘bioactive' TGF-β protein (hereafter aTGF-β). TGF-β factors are produced by numerous cell types^42^, including megakaryocytes and stromal cells^35^, into the BM microenvironment where it is activated notably by schwann cells^43^. Interestingly, HSCs do also produce TGF-β (ref. 44). Although the level of aTGF-β proteins increases in the BM microenvironment following 5FU-induced myeloablation^36^, aTGF-β did not increase in BM following irradiation/transplantation (Fig. 5a). Moreover, aTGF-β levels in BM fluid were similar between the genotypes after transplantation (Fig. 5b). Instead, aTGF-β drastically increased in 2T-WT LSK-SLAM but not in 2T-p190-B^−/−^ LSK-SLAM at 4 weeks and up to 4 months following engraftment relative to 0T LSK-SLAM (Fig. 5c,d). We used an antibody that specifically recognizes aTGF-β but not the latent inactive form (Supplementary Fig. 7). Levels of latent TGF-β1 were however similar (Fig. 5e). p190-B-deficient LSK-SLAM remained responsive to rTGF-β1 *in vitro*, as rTGF-β1 treatment promoted their asymmetric divisions (Fig. 5f). Hence, p190-B loss limits TGF-β signalling by preventing aTGF-β production in HSPCs following engraftment.

### Overexpression of aTGF-β decreases HSPC activity

**Figure 6:**
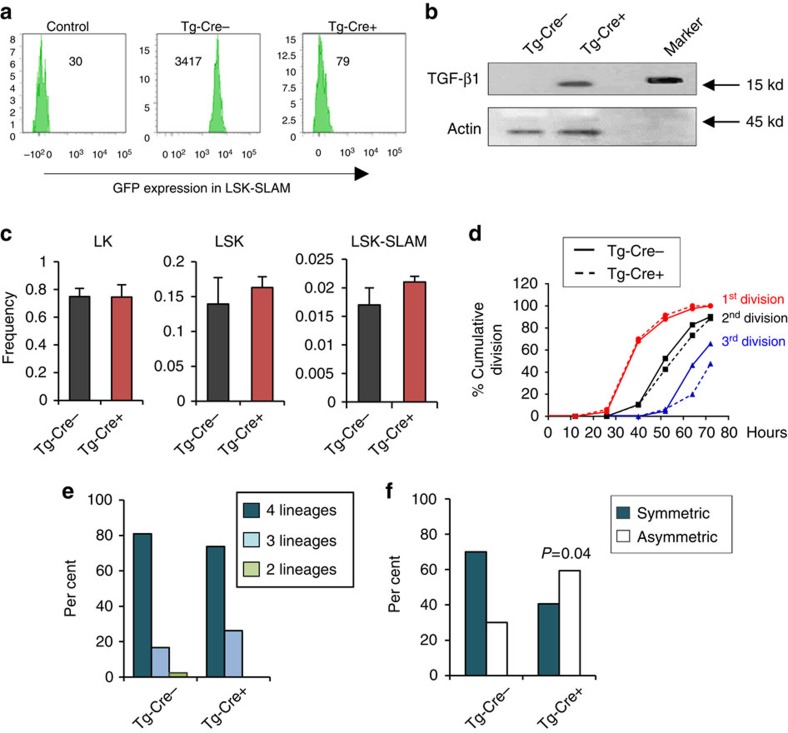
Over expression of aTGF-β promotes HSPC differentiation. Mice. transgenic for MxCre+/Flox-EGFP-STOP-aTGFb [Tg-Cre+] and MxCre-/Flox-EGFP-STOP-aTGFb [Tg-Cre−] were analysed 3–4 weeks after poly-IC injection. All data are from at least 2 independent experiments. (**a**) Histograms of flow cytometry analysis of EGFP expression in LSK-SLAM cells. (**b**) Western blot analysis of LSK cells showing overexpression of aTGF-β (expected size for aTGF-β is around 15–20 kd). Actin was used as an isnternal control. (**c**) Bar graphs show frequency of LSK-SLAM, LSK and LK population in BM (mean±s.e.m.; *n*=9 mice per group). (**d**) Cell division kinetics. Single LSK-SLAM cells from each group were cultured with medium containing SCF+TPO. Wells were examined every 12 h to determine division kinetics (*n*=30–50 cells per division per group, two independent experiments). (**e**) Single cell multilineage differentiation assay of LSK-SLAM cells isolated from each group. Single cells were isolated and cultured with serum and multiple cytokines to induce terminal myeloid differentiation, for 14 days. Clones were analysed as in Fig. 1. Bar graph shows per cent of clones containing 4, 3 and 2 lineages (*n*=40–80 clones per group, two independent experiments). (**f**) *In vitro* paired daughter cell assay performed with LSK-SLAM cells. Single LSK-SLAM cells were cultured with SCF+TPO for one division. Daughter cells were separated and further cultured individually with serum and cytokines to assess multilineage differentiation potential of daughter cells, as in Fig. 1. Bar graph shows frequency of asymmetric and symmetric divisions (*n*=20–30 pairs per group, two independent experiments). *P* value was calculated using fisher exact 2 × 2 contingency table.

To further assess whether increased aTGF-β production in HSPCs affects their functions, we used transgenic *Tg-b1glo*^*+/flox*^ mice that overexpress aTGF-β1 under a ubiquitous promoter upon Cre recombinase^45^, crossed with Mx-Cre (hereafter *Tg-Cre*^*+*^, and control *Tg-Cre*^−^). The transgene TGF-β1 cDNA is mutated to prevent the assembly of the latent complex, such that when expressed the exogenous TGF-β1 protein is constitutively in a bioactive form. Its expression is blocked by an intervening floxed EGFP gene. Upon Cre recombinase, EGFP is no longer expressed, but aTGF-β1 is expressed^45^. Loss of EGFP expression in LSK-SLAM from polyIC-treated *Tg-Cre*^*+*^ mice was confirmed by flow cytometry analysis (Fig. 6a). Overexpression of TGF-β in *Tg-Cre*^*+*^ LSK cells was confirmed by immunoblot (Fig. 6b); the size of the TGF-β1 band is similar to what is expected for endogenous aTGF-β. Since exogenous aTGF-β is released in the BM fluid of *Tg-Cre*^*+*^ mice (Supplementary Fig. 8B), making assessment on HSPC functions *in vivo* complicated by multiple confounding factors including effects from the BM microenvironment and all hematopoietic lineages, LSK-SLAM were isolated 3 weeks after poly-IC injection and used for *in vitro* experiments. Still, at this time, overexpression of aTGF-β did not alter the frequencies of LSK-SLAM, LSK and LK (Fig. 6c), or change cell cycle and survival of LSK-SLAM and LSKCD48^−^, respectively (Supplementary Fig. 8C,D). Interestingly, the rate of first division of single *Tg-Cre*^*+*^ LSK-SLAM *in vitro* was similar to control, although the third cell division was slower (Fig. 6d). In single cell assays, the frequencies of nemM clones generated *in vitro* by *Tg-Cre*^−^ and *Tg-Cre*^+^ LSK-SLAM were similar (Fig. 6e). However, in the *in vitro* paired daughter cell assay, symmetric divisions of single *Tg-Cre*^+^ LSK-SLAM were drastically reduced (Fig. 6f). These results suggest that overexpression of aTGF-β is sufficient to alter the outcome of LSK-SLAM divisions and may favour more rapid HSPC differentiation *in vitro*.

### P190-B controls aTGF-β via reactive oxygen species

**Figure 7:**
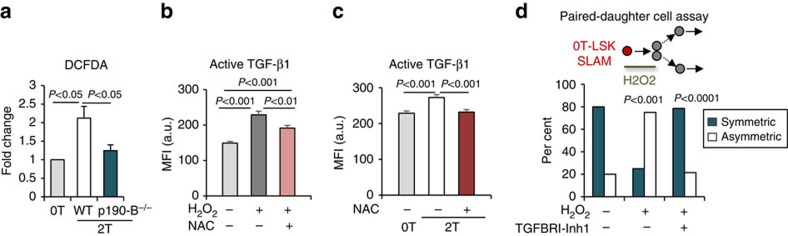
Increase in reactive oxygen species changes HSPC functions via TGF-β1 signalling. (**a**) ROS detected by DCFDA staining in LSKCD48^−^ cell population from BM of 0T, 2T-WT and 2T-p190-B^−/−^ mice 5–7 weeks following transplant, analysed by flow cytometry. Data are presented as fold change in mean fluorescence intensity relative to 0T. (*n*=8–9 mice from three independent experiments (mean±s.e.m., two-tailed unpaired *t*-test). (**b**) Detection of aTGF-β by immunofluorescence staining: Effect of H_2_O_2_ and H_2_O_2_ +NAC treatments *ex vivo* for 12 h. Bar graph shows mean fluorescence intensity in arbitrary unit (mean±s.e.m.; 40–50 cells per experiment were analysed in each group, three independent experiments, two-tailed unpaired *t*-test). (**c**) 2T-WT mice were treated with NAC for 5–8 weeks following transplantation. 2T-WT mice not treated with NAC were used as controls. LSK-SLAM cells were immuno-stained for aTGF-β. Bar graph shows mean fluorescence intensity in arbitrary unit (mean±s.e.m.; 40–50 cells per experiment were analysed in each group, three independent experiments, two-tailed unpaired *t*-test). (**d**) Effect of H_2_O_2_ on 0T LSK-SLAM division output using the *in vitro* paired daughter cell assay. Single LSK-SLAM cells isolated from 0T-WT mice were treated with H_2_O_2_ or with H_2_O_2_+TGFBRI-Inh1 for the duration of one division. Daughter cells were analysed as in Fig. 1. Bar graph shows frequency of asymmetric and symmetric divisions (*n*=28 pairs per group, two independent experiments). *P* values were calculated by Fisher exact 2 × 2 contingency table by comparing per cent of symmetric and asymmetric divisions of H_2_O_2_ versus control, and of H_2_O_2_+TGFBRI-Inh versus H_2_O_2_ treatments.

Since the levels of latent TGF-β were similar between the groups, p190-B likely controls maturation of aTGF-β in HSCs. We examined the role of ROS because ROS can directly oxidize latent TGF-β, subsequently releasing aTGF-β^46^,^47^, and is known to limit HSC lifespan^48^. Consistently, ROS levels were significantly elevated in 1T or 2T WT LSKCD48^−^ cells compared with 0T WT LSKCD48^−^ cells, but not in 2T-p190-B^−/−^ LSKCD48^−^ (Fig. 7a, Supplementary Fig. 9A). Strikingly, treating 0T LSK-SLAM with reagents known to increase ROS, that is, hydrogen peroxide (H2O2) or rotenone, a mitochondrial complex I inhibitor, increased aTGF-β levels in LSK-SLAM *in vitro*. Co-treatment with the ROS scavenger N-acetyl-L-cysteine (NAC) confirmed this increase was ROS dependent (Fig. 7b, Supplementary Fig. 9B). *In vivo*, treating 2T-WT mice with NAC during 5 weeks following transplant reduced levels of aTGF-β proteins and p-smad2 in LSK-SLAM (Fig. 7c, Supplementary Fig. 9C). *In vitro*, H2O2 treatment promoted asymmetric division of 0T LSK-SLAM (Fig. 7d). Interestingly, this effect was completely reverted when the cells were also treated with SB431542 (Fig. 7d). These findings suggest a potential link between ROS and TGF-β on HSPC functions.

### p38MAPK pathway mediates TGF-β effect on HSPC activity

**Figure 8:**
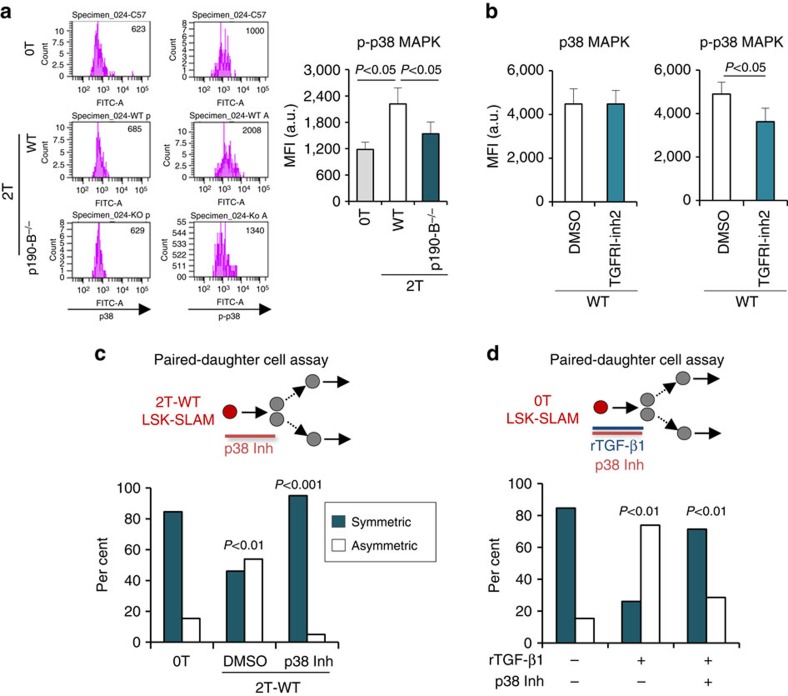
p38^MAPK^ pathway mediates TGF-β effect on HSPC fate decisions. (**a**) Flow cytometry analyses of p38^MAPK^ and p-p38^MAPK^ levels in LSK-SLAM from 0T, 2T WT and p190-B^−/−^ mice (*n*=7 independent samples, from at least two independent experiments). (**b**) Flow cytometry analyses of p-p38^MAPK^ levels in LSKCD48^−^ cells from 2T-WT mice that were treated with TGF-β RI kinase inhibitor II or DMSO *in vivo* (*n*=9 independent samples from 2 independent experiments; mean±s.e.m., two-tailed unpaired *t*-test). (**c**) Effect of p38^MAPK^ inhibitor on 2T-WT LSK-SLAM division output using the *in vitro* paired daughter cell assay as in Fig. 1. Single LSK-SLAM cells isolated from 2T-WT recipients were treated with p38^MAPK^ inhibitor for one division; daughter cells were analysed as in Fig. 1. Bar graph shows per cent of symmetric and asymmetric divisions (*n*=20–26 pairs from three independent experiments). *P* values were calculated by Fisher exact 2 × 2 contingency table. (**d**) *In vitro* paired daughter cell assay performed with LSK-SLAM cells isolated from 0T mice and treated with rTGF-β1 alone or rTGF-β1+SB203580. Bar graph shows per cent of symmetric and asymmetric divisions (*n*=21–23 pairs in two independent experiments). *P* values were calculated by Fisher exact 2 × 2 contingency table.

TGF-β can signal via canonical Smad transcription factors, and non-canonical pathways—for example, TRAF6/TAK1/p38^MAPK^ and Par6/Rho-ROCK^30^,^49^. We focused on p38^MAPK^ because Smad2 phosphorylation was similar between the genotypes following transplantation (Supplementary Fig. 10A). Further, p38^MAPK^ can be regulated by p190-B in other cells^24^ and limits HSC self-renewal^48^,^50^. *In vitro*, p38^MAPK^ activity can be triggered by rTGF-β1 in an ALK5-dependent manner (Supplementary Fig. 10B). We found p38 phosphorylation (pp38) increased in WT LSK-SLAM (Fig. 8a) following irradiation and transplantation^48^, but not in 2T-p190-B^−/−^ LSK-SLAM (Fig. 8a). Similar results were obtained in cells isolated from primary recipients (Supplementary Fig. 10C). Remarkably, pp38 levels were reduced in LSK-SLAM when mice were treated with TGFBRI inhibitor2 (Fig. 8b) or with NAC following transplantation (Supplementary Fig. 8D)^48^. As previously noted, NAC treatment also lowered levels of aTGF-β (Fig. 7c), suggesting a possible link between ROS, aTGF-β and pp38 *in vivo*. *In vitro*, SB203580, a p38^MAPK^ activity inhibitor, completely rescued symmetric division of 2T-WT LSK-SLAM in the *in vitro* paired daughter cell assay (Fig. 8c) and it prevented effect of rTGF-β1 on 0T LSK-SLAM divisions since single LSK-SLAM treated with rTGF-β1 plus SB203580 generated two nemM daughter cells at a frequency similar to LSK-SLAM treated with vehicle, compared with rTGF-β1 treatment alone (Fig. 8d). This suggests p38^MAPK^ activity mediates TGF-β effects on HSPC differentiation *in vitro*.

To further examine a link between p190-B, rTGFβ1 and p38^MAPK^ in HSC activity, pooled p190-B^−/−^ LSK-SLAM cells, isolated from primary transplanted recipients (Table 1) or fetal livers (Supplementary Table 3), were treated *ex vivo* with rTGF-β1 either in the presence or absence of SB203580, for the duration of one division. HSC activity was examined in competitive transplantation using near-limiting dilution settings. Such culture conditions did not alter cell numbers. But, LT-HSC activity of pooled p190-B^−/−^ LSK-SLAM cells cultured with rTGF-β1 was significantly reduced (30% of transplanted mice showing greater than 1% contribution to both myeloid and lymphoid lineages in PB versus 56% from non-treated cultures). This effect was prevented by addition of SB203580 (65% mice were engrafted (Table 1 and Supplementary Table 3). Of note, the mice showing <1% donor-cell contribution in at least one lineage had lost durable myeloid cell contribution, which indicates the donor cell population exhibited ST-HSC activity. Hence, rTGF-β1 can alter HSC activity via p38^MAPK^ signalling pathway *in vitro*. These findings suggest that p190-B loss may favor the likelihood of HSC stemness inheritance during *in vitro* divisions, by preventing autonomous activation of TGF-β-p38^MAPK^ axis.

**Figure 9:**
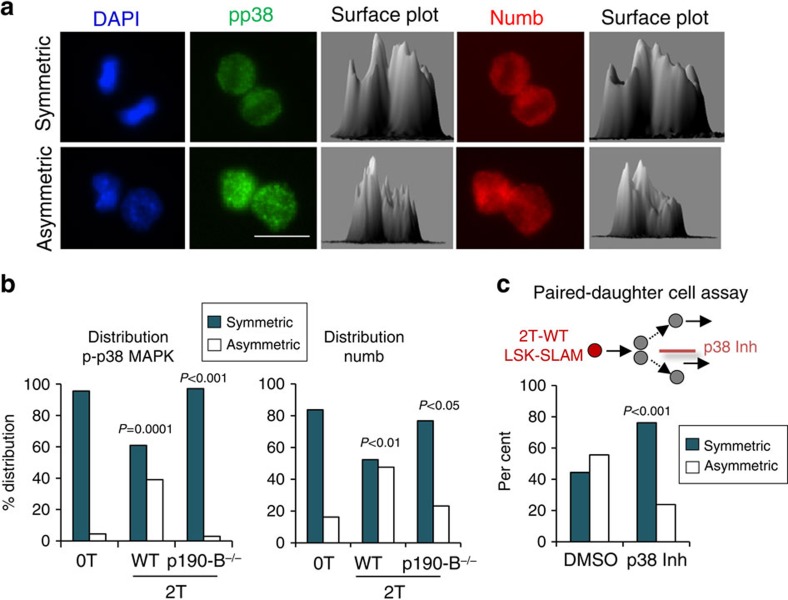
Asymmetric inheritance of p-p38^MAPK^ and numb in 2T WT-LSK-SLAM. (**a**) LSK-SLAM cells from 0T, 2T WT and 2T-p190-B^−/−^ mice were cultured for 40 h and stained for p-p38^MAPK^ (in green), Numb (in red) and DAPI (inblue), scale bar, 10 μm. 3D plots using ImageJ software represent distributions of p-p38^MAPK^ and Numb in daughter cells. (**b**) Bar graphs represent per cent of symmetric and asymmetric distribution of p-p38^MAPK^ and Numb in mitotic cells. 35–40 mitotic cells from three independent experiments were analysed. *P* values were calculated by Fisher exact 2 × 2 contingency table comparing 2T-WT to control and 2T-p190-B^−/−^ to 2T-WT. (**c**) Effect of p38^MAPK^ inhibitor on daughter cell multilineage differentiation potential. Single 2T-WT LSK-SLAM cells were cultured with SCF+TPO for one division. Paired-daughters were separated and individually cultured with serum and multiple cytokines for mature differentiation for 14 days in the presence or absence of p38^MAPK^ inhibitor. Resultant clones were analysed as in Fig. 1 (*n*=20 pairs in two independent experiments). *P* values were calculated by Fisher exact 2 × 2 contingency table by comparing per cent of symmetric and asymmetric divisions of p38^MAPK^ inhibitor versus DMSO.

Because asymmetric cell division is controlled by asymmetric inheritance of cell fate determinants, inheritance of pp38 and Numb by daughter cells was examined. Numb, a conserved cell fate determinant, can be asymmetrically distributed during HSPC division^51^ and is a marker of differentiation^39^. Remarkably, pp38 was symmetrically distributed to daughter cells of 0T and 2T p190-B^−/−^ LSK-SLAM along with low levels of Numb. However, pp38 was asymmetrically inherited by daughter cells of 2T WT LSK-SLAM (Fig. 9a,b). In 70% of asymmetric divisions, the daughter cells receiving high pp38 also received high Numb whereas the daughter cells inheriting low pp38 also had low Numb, suggesting high correlation between pp38 and Numb distributions (Fig. 9a,b). In the remaining divisions, Numb was found equally distributed to daughter cells whereas pp38 distribution was asymmetric. Since addition of p38^MAPK^ inhibitor after daughter cell separation changed the multilineage potential of the daughter cells (Fig. 9c), asymmetric distribution of pp38 is likely important to dictate the level of differentiation of the daughter cells. These findings suggest association between pp38 segregation and HSPC multilineage potential *in vitro*. Since low p38^MAPK^ signalling intensity also correlated with maintenance of HSPC activity independent on cell cycle progression, p38^MAPK^ pathway may play important roles in HSPC commitment to differentiation. Together, p190-B-TGF-β-p38^MAPK^ network represents a novel regulatory pathway of HSPC activity independent of cell cycle progression.

## Discussion

The factors that control HSPC fate decision remain unclear. The paired daughter cell assay *in vitro* allows quantitative assessment of HSPC lineage commitment during active division, and may thus provide insights into how HSPC fate decisions are regulated^6^,^7^,^8^,^9^,^15^. Although information on lymphoid potential is missing and although HSC self-renewal cannot be assessed, loss of multi-lineage myeloid potential correlates with loss of long-term repopulation potential^52^,^53^. Our findings suggest the existence of a signalling network, p190-B-TGF-β-p38^MAPK^ activity that controls HSPC activity independent of cell proliferation—and may control a fate decision leading to HSC accelerated differentiation during division.

HSC fate can be instructed by cytokines. Stem cell factor dose-dependently maintains HSC self-renewal divisions *in vitro*; and can instruct HSC fate even before the first cell division^6^. Nerve growth factor and Collagen 1 in combination with SCF and IL-11 support HSC activity inheritance through divisions^7^. Lnk negatively regulates HSC self-renewal divisions downstream of thrombopoietin^16^. These fate decisions can depend on intrinsic polarity pathway^39^. In this case, polarity cues drive the asymmetric distribution of cell fate determinants to each daughter cell—leading to distinct cell fate. The conserved pathway of asymmetric division involves the canonical polarity pathway Par/aPKC, which determines the asymmetric inheritance of the cell fate determinant Numb—an inhibitor of NOTCH signalling^39^. Although Numb can be asymmetrically distributed to daughter cells in HSPCs^51^, HSC functions are preserved in absence of aPKC expression^54^, suggesting other pathways also contribute to asymmetric divisions. Other factors, including peroxisome proliferator-activated receptor δ (PPAR-δ)–fatty-acid oxidation (FAO) pathway^8^, the transcription factor Satb1 (ref. 55) or Musashi-2 (ref. 56), were shown to be asymmetrically partitioned during HSPC division. More recently, Lis1, a canonical regulator of spindle orientation during division, was shown to be important for HSC integrity^57^. However, most of these factors also altered HSC quiescence/proliferation. The Sauvageau group reported that the endocytic protein Ap2a2 is asymmetrically distributed during HSPC division, and expression of Ap2a2 enhances HSC activity, independent of HSC proliferation, making it a rarely identified ‘fate determinant' in HSC^17^. Our findings suggest that p190-B and downstream regulatory elements abrogate HSPC activity through divisions *in vitro*, and alter HSPC functions *in vivo* in absence of any effect on cell survival and proliferation. Hence, balancing HSPC fate decision to commit to differentiation may be a mechanism by which this signalling network controls HSC engraftment *in vivo*. p190-B is also known to regulate adipogenesis versus myogenesis fate decisions of fibroblasts and mesenchymal cells^25^. We recently showed that it controls mesenchymal stem cell fate specification to adipocyte and osteoblast lineages both *in vitro* and *in vivo*. These decisions are important for the development of a functional mesenchymal stem cell niche during ontogeny^58^, and for the development of bones and fat. Hence, p190-B may be a major, yet under-appreciated, regulator of somatic stem cell fate specification.

Most intriguingly is the finding that TGF-β-p38^MAPK^ signalling pathway mediates this process. We found that TGF-β/p38^MAPK^ signalling increases in HSPCs following transplantation. When TGF-β/p38^MAPK^ signalling pathway is high following engraftment, HSPCs appear to commit to differentiation, at least as seen using *in vitro* assays. When TGF-β/p38^MAPK^ signalling remains low, either due to loss of p190-B expression or the use of TGFBRI pharmacological inhibitors, HSC self-renewal activity is maintained. The use of pharmacological inhibitors was essential to demonstrate this. It allowed us a transient and reversible inhibition of TGF-β signalling, hence circumventing pleiotropic action of TGF-β on other hematopoietic cells. Interestingly, there was a clear association between the asymmetric cell shape of transplanted HSPCs, the asymmetric distribution of pp38 itself to their daughter cells and the fact that these daughter cells exhibited distinct differentiation stages, at least *in vitro*. Asymmetric pp38 distribution was positively correlated with asymmetric distribution of Numb. Further, inhibition of p38^MAPK^ activity in the daughter cells could reverse their potential into multipotent progenitors *in vitro*. These findings together suggest that the level of pp38 can dictate HSPC state and that a daughter cell receiving high levels of pp38 may be more prone to commit to differentiation. Noteworthy, p38^MAPK^ activity is important for asymmetric self-renewal division of satellite cells during injury-induced skeletal muscle repair. In these cells, p38^MAPK^ activity is activated in one daughter cell only, inducing MyoD expression and tissue regeneration. The absence of p38^MAPK^ activation in the other daughter cell prevents MyoD induction, thus enabling self-renewal^59^. Hence, fluctuations in signalling intensity and p38^MAPK^ activity are perhaps *bona fide* fate determinants in HSPCs. Our study is an important extension to the existing notion that different signalling intensity distinctly supports HSC state^6^. This is in line with the (re)emerging notion that, after all, cytokines may have instructive roles in HSC fate decision to commit or not to differentiation^60^.

Mechanistically, it seems that HSCs intrinsically control the levels of TGF-β signalling by producing the active form of TGF-β. Upon serial transplantation, WT HSCs acquired long-lasting expression of aTGF-β. Enhancement of aTGF-β in HSCs was responsible for HSC activity changes occurring during hematopoietic regeneration. This is best supported with the observation that overexpression of aTGF-β using genetic approach is sufficient to promote HSPC differentiation *in vitro* in absence of cell cycle progression changes. ROS appears to be one mechanism of aTGF-β maturation in cells, perhaps via direct oxidation of the pro-TGF-β into aTGF-β, as seen in other cells^46^,^47^. Interestingly, this places ROS upstream of TGF-β in HSPC fate decisions. Recently, hypoxia and autocrine TGF-β were shown to promote human CD34+ quiescence, although there was no evidence that hypoxia directly controls secreted TGF-β (ref. 61). In the hematopoietic system, TGF-β is best known for its potent growth inhibitory effect^62^. *In vivo*, TGF-β can be secreted and activated in the BM microenvironment, and acts in a paracrine manner to induce HSC hibernation^43^. In addition, bioactive TGF-β proteins can increase in the BM microenvironment following 5FU-induced myeloablation, and impair HSC regeneration by limiting their proliferation^36^. TGF-β seems important during the aging of the hematopoietic system, distinctly affecting the proliferation of myeloid versus lymphoid-biased subsets of HSCs^13^. On the other hand, TGFBRI deficiency revealed that HSC functions are maintained over serial transplantation without ALK5 activity^63^. We found TGF-β can control HSC activity independent of cell proliferation. These seemingly contradictory findings are likely the reflection of multifaceted functions of TGF-β. This may be dosage dependent. TGF-β can induce or suppress proliferation in a concentration-dependent manner^13^. We show that rTGF-β alters HSC activity at low concentration. It also may be due to an autocrine-specific action, and/or the use of different downstream signalling activity. As discussed above, in our model, bioactive TGF-β proteins are produced by HSCs. And, TGF-β may use p38^MAPK^ signalling pathway instead of the canonical smad transcription factors^64^. An autocrine TGF-β pathway that acts independent of smads is not without precedent. In a cancer cell line Mv1Lu, autocrine TGF-β acts through JNK and p38^MAPK^ (ref. 65). Hence, we propose that HSCs autonomously produce aTGF-β to modulate TGF-β-p38^MAPK^ signalling and alter HSPC activity during stress-induced regeneration. This is controlled by p190-B. Interplay between Rho GTPase signalling and autocrine TGF-β-p38^MAPK^ signalling may represent an early signal activated during HSC division to balance self-renewal and differentiation during HSC regeneration. Hence, our data reveal what may be a novel function for TGF-β in HSC self-renewal activity as fate determinant to modulate HSC activity.

## Methods

### Reagents

Inhibitors used: SB431542 (inhibitor of TGF-β1 receptor ALK5, Cayman chemical [TGFBRI inhibitor 1]), TGF-β RI kinase inhibitor II (Calbiochem, [TGFBRI inhibitor 2]), SB203580 (p38^MAPK^ inhibitor, Calbiochem), PKI (6–22) amide (protein kinase inhibitor, Santa Cruz Biotech). CHIR-9902 (GSK3β inhibitor), LDN193189 (BMP inhibitor), Cyclopamine (hedgehog signalling pathway inhibitor) were all from Selleck Chemicals.

### Mice model

p190-B RhoGAP^+/−^ mice (backcrossed into C57BL/6J), B6.SJL-PtrcaPep3b/BoyJ (B6.BoyJ, CD45.1^+^) congenic mice. To study effect of over-expression of TGFβ1 on HSCs; transgenic Tg-b1glo^+/Flox^ mice (FVB; Jackson Lab)^45^ were crossed with Mx1-Cre to generate MxCre^+^; Tg-b1glo^+/Flox^ [Tg-Cre^+^] and MxCre^−^; Tg-b1glo^+/Flox^ mice [Tg-Cre^−^]. To activate cre recombinase, polyIC was injected intraperitoneally to mice (10 μg g^−1^ of body weight; three injections—every alternate day for 1 week). All animals were bred in house in pathogen-free environment. All studies were conducted with a protocol approved by the Animal Care Committee of Cincinnati Children's Hospital Medical Center.

### Serial competitive repopulation assays

To examine the role of p190-B loss during serial competitive transplantation, embryonic day 14.5 p190-B^−/−^ fetal livers (FLs) and WT littermates (0.3 × 10^6^ FL cells, CD45.2^+^) were transplanted along with competitor cells (1.7 × 10^6^ BM, CD45-1^+^) into lethally irradiated congenic recipient mice (CD45.1^+^). Four months following primary transplant (1T), 10^6^ BM cells from 1T mice were injected in secondary (2T) lethally irradiated congenic recipient mice (CD45.1^+^). Un-transplanted (0T) mice were used as control.

### *In vitro* paired daughter cell assay

*In vitro* paired daughter cell assay was performed as described before^9^,^15^. Briefly single LSK-SLAM cells were sorted into 96-well plate. Single cells were visually confirmed under light microscope and cultured in serum free Stemspan medium (Stem Cell Technology) supplemented with murine SCF and murine TPO (100 ng ml^−1^, each, Peprotech) during the first cell division only. After the first division, daughter pairs were separated and individually cultured in Iscove's Modified Dulbeco's Medium (IMDM) containing 10% fetal bovine serum (Omega) and a cocktail of cytokines allowing for myeloid differentiation (murine SCF, murine TPO, human G-CSF (each 20 ng ml^−1^), murine IL-3 (50 ng ml^−1^) and EPO (4 U ml^−1^; Espogen)) for 14 days. Paired-clones were harvested and used for cytospin preparation. Cells of various lineages were identified based on their morphology after Diff-quick staining (Siemens). Clones were examined for the presence of neutrophils (n), erythroid cells (e), macrophages (m) and megakaryocytes (M; nemM). In some experiments, single LSK-SLAM cells were also incubated with or without TGFBRI inhibitors 1 or 2 (10 μM) or p38^MAPK^ inhibitor (10 μM) or GSK3β inhibitor; (3 μM) or human recombinant TGF-β1 10 pg ml^−1^ (Peprotech) or H2O2 (50 μM) for the duration of the first division only. Daughter cells were separated and individually cultured in serum and cytokines without inhibitors. Clones were analysed as above.

### Single cell multi-lineage differentiation potential assay

Single LSK-SLAM cells were cultured in Stemspan medium containing murine SCF and murine TPO (each 100 ng ml^−1^) for the first 48 h and then in IMDM containing 10% fetal bovine serum, murine SCF, TPO, G-CSF, EPO and IL-3. Cytokine concentrations were similar as used for paired daughter cell assay. Clones derived from LSK-SLAM were harvested at 14 days of culture, and examined for the presence of myeloid lineages (neutrophil, erythroid, macrophage and megakaryocyte).

### Cell division kinetics

Single LSK-SLAM cells were isolated in 96-well plates and cultured in Stemspan medium containing murine SCF and murine TPO (each 100 ng ml^−1^). Wells were visually examined to count numbers of cells per well every 12 h for 72 h and determine division kinetics. A first division was scored when two cells could be observed; a second division was scored if three or four cells were observed. Data were expressed as per cent cumulative division at every time interval.

### Effect of TGF-β inhibitor *in vivo*

2T mice transplanted with WT cells were injected subcutaneously twice daily with TGFBRI inhibitor 2 (100 μl of 1:10 dilution of 3.4 mM inhibitor in PBS) or dimethyl sulfoxide (DMSO; vehicle control)^32^ for 4 weeks. Mice were used for experiments one or two weeks later. PB was analysed for donor-cell chimerism and donor-cell derived (CD45.2^+^) myeloid and lymphoid lineage differentiation. BM cells of these mice were used for *in vitro* assays and for limiting competitive repopulation assay to measure HSC frequency. For the latter, 1 × 10^6^ or 5 × 10^6^ BM cells were mixed with 0.25 × 10^6^ BM CD45.1+ cells and injected into tertiary recipients (CD45.1^+^).

### *Ex vivo* culture and transplantation

LSK-SLAM cells (300 or 500) isolated from 1T mice or fetal livers were cultured in serum free Stemspan medium containing murine SCF and murine TPO (100 ng ml^−1^ each) for 48 hours in the presence of SB431542 (10 μM), or rTGF-β1 or rTGF-β1+ SB203580 (10 μM) or equivalent DMSO, depending on the experiments. Cultured cells were mixed with 0.2 × 10^6^ BM CD45.1+ cells and injected into lethally irradiated mice (CD45.1^+^).

### Flow cytometric analyses

The following antibodies were used for flow cytometric analyses (unless specified all antibodies were from BD biosciences): anti-CD45.1-PE (A20), anti-CD45.2-FITC or—Percpcy5.5 (104), anti-CD11b-FITC (M1/70), anti-Gr1-PE (RB6-8C5, ebioscience), anti-B220-APC (RA36B2), anti-CD4- FITC (RM4-5), anti-CD8a-PE (53-6.7), anti-Sca-1-PECy7 (D7), anti-c-Kit-PE or—APC (2B8), anti-CD48-FITC (HM48-1) and anti CD150-APC (9D1, ebioscience). For lineage negative population we used: anti-CD11b, anti-Gr1, anti-B220, anti-Ter119, anti-CD3, anti-CD5-biotin-labeled followed by streptavidin APCCy7. All antibodies were used at a 1:100 dilution.

For intracellular detection of p38^MAPK^ and phosphorylated p38^MAPK^, BM cells were first stained for LSK-SLAM. Cells were then fixed and permeabilized using cytofix/cytoperm buffer (BD Bioscience) as per manufacturer's protocol. The cells were stained with purified rabbit anti-p38^MAPK^ or anti-pp38^MAPK^ antibodies (1 μl in 100 μl; Cell Signaling) followed by donkey anti-rabbit AF488 (0.4 μl in 100 μl; Invitrogen).

To detect intracellular ROS, BM cells were first stained for LSKCD48 and then incubated with 2′,7′ dichlorofluorescin diacetate (DCFDA) for 30 min at 37 °C. The cells were analysed by flow cytometry immediately.

Side population (SP) staining, was carried as described previously (10). Briefly, low density BM cells were first stained with Hoechst 33342 (5 μg ml^−1^; Sigma) for 90 min at 37 °C with or without Fumitremorgin C (10 μM; Enzo Life Sciences) and then stained for LSK-SLAM surface markers. Fluorescence activated cell sorting was performed on FACS Aria and analysis was performed on FACS Canto or LSR II (BD).

### Colony forming unit assay

Cultured LSK-SLAM cells were plated in semi-solid methyl cellulose medium containing serum and cytokines (IL3, SCF, G-CSF 100 ng ml^−1^ each and Epo 4 U ml^−1^) and incubated at 37 °C. Differential colonies were scored at day 7.

### Cell cycle analysis

BM cells from serially transplanted mice were stained for cell-surface markers, fixed, permeabilized and then incubated with Hoechst 33342 (10 μg ml^−1^, Invitrogen) and Pyronin Y for 30 min at 37 °C (1 μg ml^−1^; Sigma-Aldrich) or Hoechst 33342 and ki67 (1:100 dilution, ebioscience) for 30 min at 4 °C in the dark.

### 5-Fluorouracil

5-Fluorouracil (5-FU;150 mg kg^−1^) was administered by intraperitoneal injection. PB differential counts and lineage reconstitution were analysed after retro-orbital bleed^19^.

### Microarray analyses

LSK-SLAM were isolated from secondary recipients of WT or p190-B^−/−^ cells. Cells were lysed in lysis buffer (Miltenyi Biotech) and samples were further processed at Miltenyi Biotech, Germany, using Agilent Whole Mouse Oligo Microarrays. Data were analysed by gene set enrichment analysis across the complete list of genes. Gene ontology analysis on top candidate genes based on Student's *t*-test analysis was performed using ToppGene Suite software. The accession number for the raw data is GSE89794.

### qPCR analyses

LSK cells were isolated from 2T WT and p190-B^−/−^ mice, and control. RNA isolation and cDNA preparation were performed as per manufacture's protocol (RNeasy micro kit, Quiagen, Superscipt III system, Invitrogen). cDNA was amplified by real time PCR using SYBR green master mix (SA Bioscience).

### Immuno-fluorescence staining

LSK-SLAM cells were isolated on retronectin coated glass slides in Stemspan medium. The cells were incubated at 37 °C for 30 min, fixed with 4% paraformaldehyde, permeabilized with 0.5% Triton X100 and blocked with 2% bovine serum albumin in PBS at room temperature. The cells were immuno-stained with a mouse anti-aTGFβ-1(1:100 dilution; R&D Systems) that detect bioactive TGFβ-1 or goat anti-hLAP/TGFβ-1 (1:100 dilution; R&D systems) to detect latent TGFβ-1 or rabbit anti-psmad2 (S465/467, 1:100 dilution; Cell Signaling). The cells were co-stained with DAPI to identify nucleus. In another experiment, 0T LSK-SLAM cells were treated with H_2_O_2_ (50 μM) alone or H_2_O_2_+N-acetyl cysteine (100 μM, NAC, Sigma-Aldrich) for 12 h and were stained with mouse anti-TGFβ-1. In another set, 0T LSK-SLAM cells were treated with rotenone (1 μM) alone and rotenone+NAC for 24 h and were stained with mouse anti-aTGFβ-1.

To study cell shape, LSK-SLAM cells from each group treated or not treated with rTGFβ-1 or SB431542 were sorted on slides, cultured for 24 h in stemspan medium with SCF and TPO (100 ng ml^−1^), then fixed and stained for F-actin (rhodamine phalloidin (Invitrogen)) and microtubules (anti-tubulin, Abcam) and DAPI.

To analyse mitotic events, 0T or 2T LSK-SLAM cells were cultured for 40 h and were stained with rabbit anti-pp38MAPK (1:100 dilution, Cell signaling) and goat anti-numb (1:100 dilution, Abcam). Secondary Ab were donkey anti-rabbit AF488 and donkey anti goat AF647, respectively. Fluorescence images were captured using Leica DMI6000 or Zeiss epifluorescence microscope equipped with ORCA-ER C4742-95 camera (Hamamatsu) driven by Openlab software. Quantification of fluorescence intensity was performed in Openlab^R^ or in ImageJ^R^ softwares. Asymmetry was defined when differences in MFI between daughter cells were at least 1.5-fold. At least 100 cells per group were analysed; at least two independent experiments were performed. 3D surface plots were generated using ImageJ^R^ software.

### Western blot

LSK cells from Tg-Cre+ and Tg-Cre− mice mice were lysed in lysis buffer. Membrane was probed with anti-TGF-β1 antibody (Abcam). Actin was used as an internal control. All uncropped western blots can be found in Supplementary Fig. 11.

### Statistical analyses

Data are expressed as mean±s.e.m. Differences were analysed by an unpaired two-tailed *t*-test. *In vitro* paired daughter cell assay data were analysed by 2 × 2 Fisher exact test contingency table and single cell multi-potential differentiation data were analysed by Chi- square 3 × 2 contingency table.

### Data availability

The authors declare that all data supporting the findings of this study are available within the article and its Supplementary information files or from the corresponding author upon reasonable request. The microarray data have been deposited in the GEO database under accession code GSE89794.

## Additional information

**How to cite this article:** Hinge, A. *et al*. p190-B RhoGAP and intracellular cytokine signals balance hematopoietic stem and progenitor cell self-renewal and differentiation. *Nat. Commun.* **8,** 14382 doi: 10.1038/ncomms14382 (2017).

**Publisher's note:** Springer Nature remains neutral with regard to jurisdictional claims in published maps and institutional affiliations.

## Supplementary Material

Supplementary Information Supplementary Figures, Supplementary Tables.

Supplementary Data 1 Differential gene expression analysis; genes that are significantly up-regulated in p190-B-KO HSPCs

Supplementary Data 2 Differential gene expression analysis; genes that are significantly down-regulated in p190-B-KO HSPCs

## Figures and Tables

**Table 1:** Effect of TGFβ on p190-B^−/−^ HSPC engraftment.

	**Chimera>1%**	**Chimera<1%**	**Total mice transplanted**	***P*** **value**
p190-B^−/−^	9	7	16	
p190-B^−/−^+rTGFβ1	4	9	13	0.0006*
p190-B^−/−^+rTGFβ1+p38 inhi	13	7	20	< 0.0001^†^

1T p190-B^−/−^ LSK-SLAM cells (300) were treated *ex vivo* with rTGF-β1 alone or rTGF-β1+SB203580 for 48 h, and transplanted in competition with 200,000 CD45.1^+^ cells. Table shows numbers of mice exhibiting more than 1% contribution to both myeloid and lymphoid lineages in PB (positive mice) and numbers of mice with less than 1% contribution in at least one lineage (negative mice) (2–3 independent experiments). *P* values were calculated by Fisher exact 2 × 2 contingency table by comparing per cent of negative and positive mice of the following groups: *p190-B^−/−^ vs p190-B^−/−^+rTGF-β1, and †p190-B^−/−^+rTGF-β1 vs p190-B^−/−^+rTGF-β1+ SB203580. Difference between the three groups was analysed by *χ*^2^ test, *P*<0.0001.
