# Supplementary material for: p190-B RhoGAP and intracellular cytokine signals balance hematopoietic stem and progenitor cell self-renewal and differentiation
Source: Nat Commun. 2017 Feb 8;8:14382. doi: 10.1038/ncomms14382 (PMC5309857; doi:10.1038/ncomms14382)
Supplement: Supplementary Information — Supplementary Figures, Supplementary Tables. [file ncomms14382-s1.pdf]

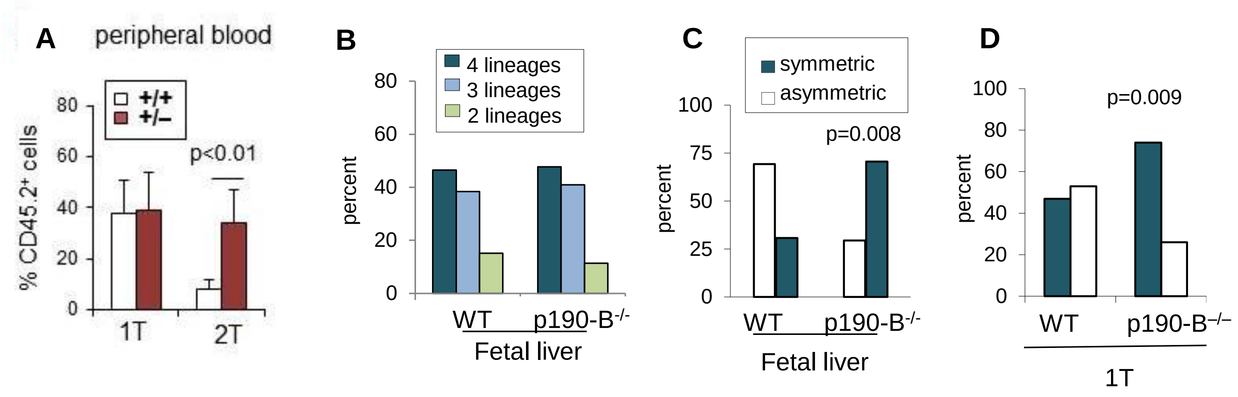

Supplementary figure 1 p190-B regulates HSPC functions independent of proliferation. (A) Serial transplantation using adult WT and p190-happloinsufficient BM cells. Data shows percent donor-cells in PB, (mean SD, unpaired t test). **(B)** Single cell multilineage differentiation assay using LSK-SLAM cells from E14.5 fetal livers. Bar graphs show percent of clones containing 4, 3 and 2 myeloid lineages (2 independent experiments; 20-80 clones analyzed). **(C-D)** In vitro paired daughter cell assay performed with LSK-SLAM cells isolated from WT and p190-B-/- fetal liver LSK-SLAM cells (C) and 1T-WT and 1T- p190-B-/- mice (D) as described in figure 1. Bar graphs represent percent symmetric and asymmetric divisions in each group. (n=25-40 pairs from 2 independent experiments). Fisher exact 2x2 contingency table was used to calculate P values.

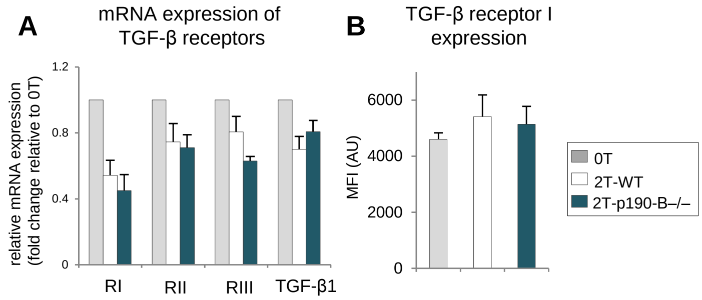

Supplementary figure 2 p190-B regulates TGF-β signaling following serial transplantation.
(A) mRNA expression analyses by qPCR of TGF-β receptors – I, II, III and TGF-β1 in LSK cells isolated from 0T, 2T-WT and 2T-p190B-/- mice. Data are normalized to β-actin and presented as fold changes relative to 0T. (mean ± SEM; n=3-5 from three independent experiments). (B) Flow cytometric analyses of TGF-β receptor I. BM cells were isolated from 0T and 2T- WT, 2T- p190-B-/- mice. Levels of TGF-β receptor I were analyzed within LSK-SLAM cell population. Histogram represents mean fluorescence intensity within the groups (mean ± SEM, n=4 independent samples).

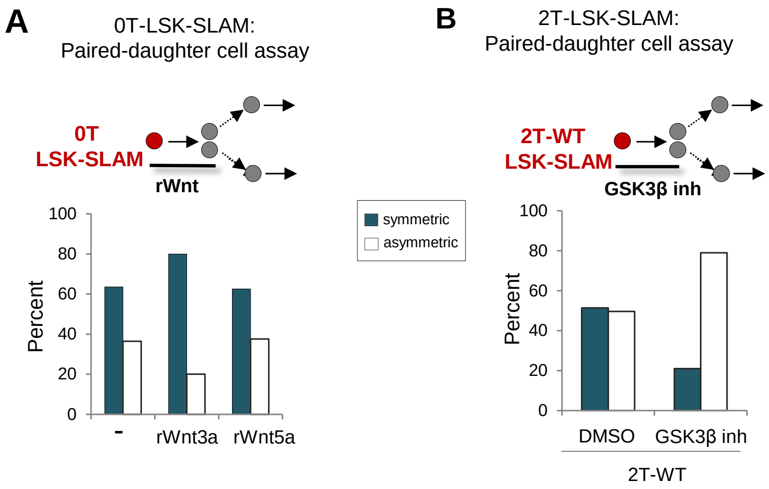

Supplementary figure 3 Effect of Wnt and GSK3β inhibitor in LSK-SLAM division output. (A) Effect of Wnt3a (100pg/ml; n=5 pairs) or Wnt5a (100pg/ml; n=8 pairs) on 0T-LSK-SLAM division output using the pair daughter cell assay as in (figure 1). **(B)** Effect of GSK3β inhibitor on 2T-WT LSK-SLAM cell division output using the pair daughter cell assay as in (B) (n=10-19 pairs). Bar graphs show percent of asymmetric and symmetric divisions in each group, from at least two independent experiments.

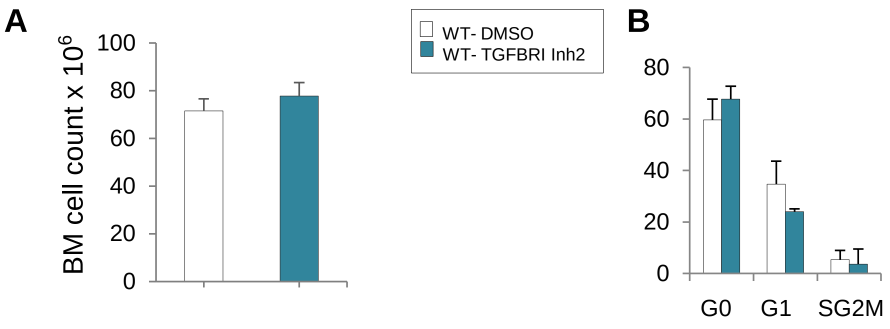

Supplementary figure 4: Inhibition of TGF-β signaling in vivo in 2T mice. Secondary recipient mice transplanted with WT cells were treated in vivo with DMSO or TGFBRI inhibitor II [TGFBRI-Inh2] for four weeks (data from 2-3 independent experiments). **(A)** BM counts (13 – 15 mice per group). **(B)** Cell cycle analysis in LSK and LSK-SLAM cells (3-4 mice per group).

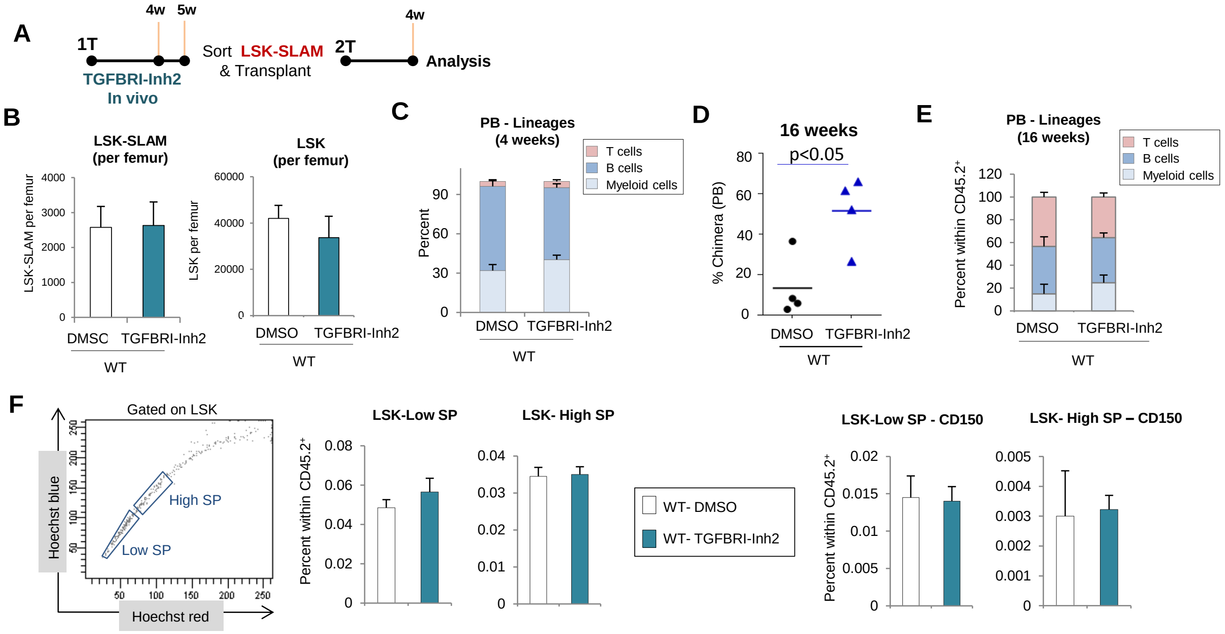

Supplementary figure 5 Inhibition of TGF-β signaling promotes HSC regeneration in vivo. (A-C) Mice were transplanted with WT cells and treated in vivo with DMSO or TGF-β RI kinase inhibitor II for four weeks, two independent experiments. **(A)** Schema of experimental design. **(B, C)** Mice were analyzed at 5 weeks post-transplant. Histograms show LSK-SLAM and LSK per femur (B) and percent mature PB blood lineage (C) (mean ± SEM; n=4 mice/group). **(D, E)** LSK-SLAM were isolated from DMSO or TGFBRI inhibitor-treated mice and transplanted into secondary recipients with competitor cells but without further treatment with inhibitor. (D) Histogram shows percent chimera in PB and (E) lineage reconstitution at16 weeks following transplant (mean ± SEM; n=4 mice per group, unpaired t test). **(F)** Dot plots of flow cytometric analyses of side population (SP) analysed in primary transplanted WT mice at 5 weeks following transplant. Low SP and high SP were analyzed in LSK cell population and CD150 expression was analyzed in LSK low SP and LSK high SP cell populations (mean ± SEM; n=4 independent samples).

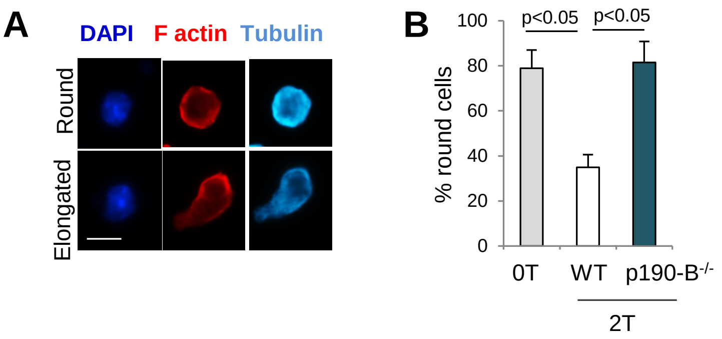

Supplementary figure 6 p190-B controls LSK-SLAM cell shape and polarity. (A) LSK-SLAM cells were isolated on chamber slides and cultured for 24 hours. Cells were fixed and immuno-stained for F-actin (in red), tubulin (in light blue) and DAPI (blue), scale bar 10μm. Representative images. **(B-D)** Quantification of cells showing round shape in 0T, WT and p190-B-/- LSK-SLAM cells **(B),** WT LSK-SLAM treated with TGFBRI inhibitor1 **(C)** and 0T LSK-SLAM cells treated with rTGF- β1 **(D)** (mean ± SEM; 3 independent experiments). P value was calculated using 2 tailed un-paired t test between the groups as indicated.

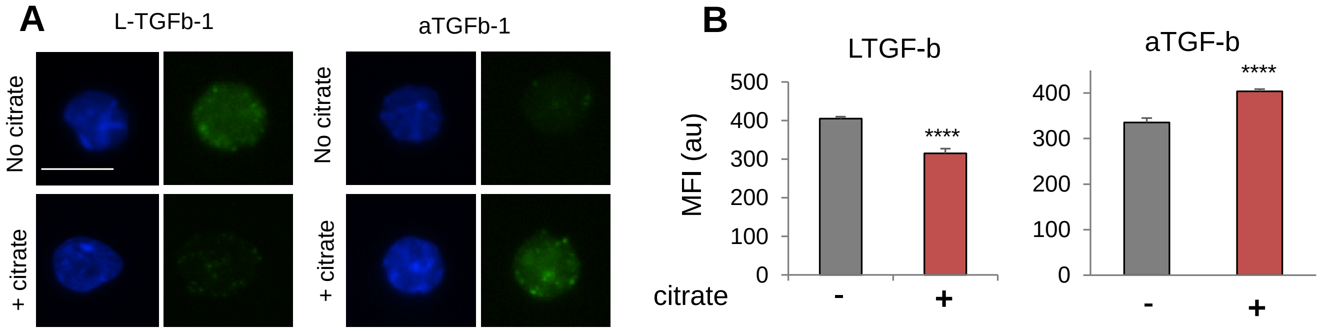

Supplementary figure 7: Validation of specificity of antibody for TGF-β1 antibody by immuno-fluorescence. **(A)** LSK cells from 0T mice were incubated in presence or absence of citrate. The cells were stained for either LTGF-β1 or aTGF-β1, scale bar 10μm**. (B)** Bar graphs show quantification of cells.**** p<0.001, 2 tailed unpaired t test.

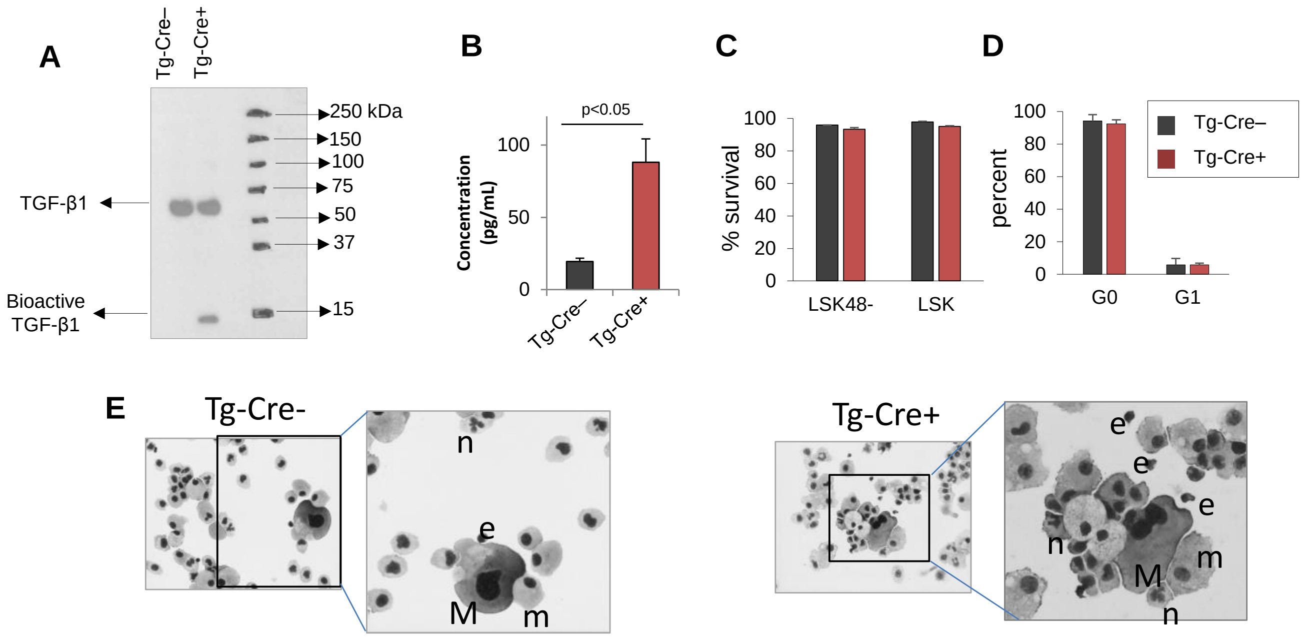

Supplementary figure 8 Over expression of aTGF-β promotes HSPC differentiation
(A) Uncropped western blots of LSK cells isolated from Tg-Cre- and Tg-Cre+ mice. Blots were probed with TGF-β1 antibody. Beta actin was used as control. **(B)** BM fluids from Tg-Cre- and Tg-Cre+ mice were collected in serum-free medium and concentration of aTGF-β was measured by ELISA (mean ± SEM; 3 independent samples, unpaired t test).**(C)** Whole BM cells from Tg-Cre- and Tg-Cre+ mice were stained for LSKCD48 and further stained with annexin V and PI to analyze apoptosis. Bar graph indicates percent live cells (annexinV- PI-) in LSKCD48- and LSK cell populations (n=3 independent samples). **(D)** Whole BM cells from Tg-Cre- and Tg-Cre+ mice were stained for LSK-SLAM and further stained with Hoechst and ki67 to analyze cell cycle status. (n=3 independent samples). **(E)** Images illustrating multi-potent clone containing 4 myeloid lineages (e: erythroid cells, n: neutrophils, m: macrophage/ monocyte, M: megakaryocyte, scale bar 10μm) generated from LSK-SLAM cells isolated from Tg-Cre- and Tg-Cre+ mice. P value was calculated using 2 tailed un-paired t test between the groups as indicated.

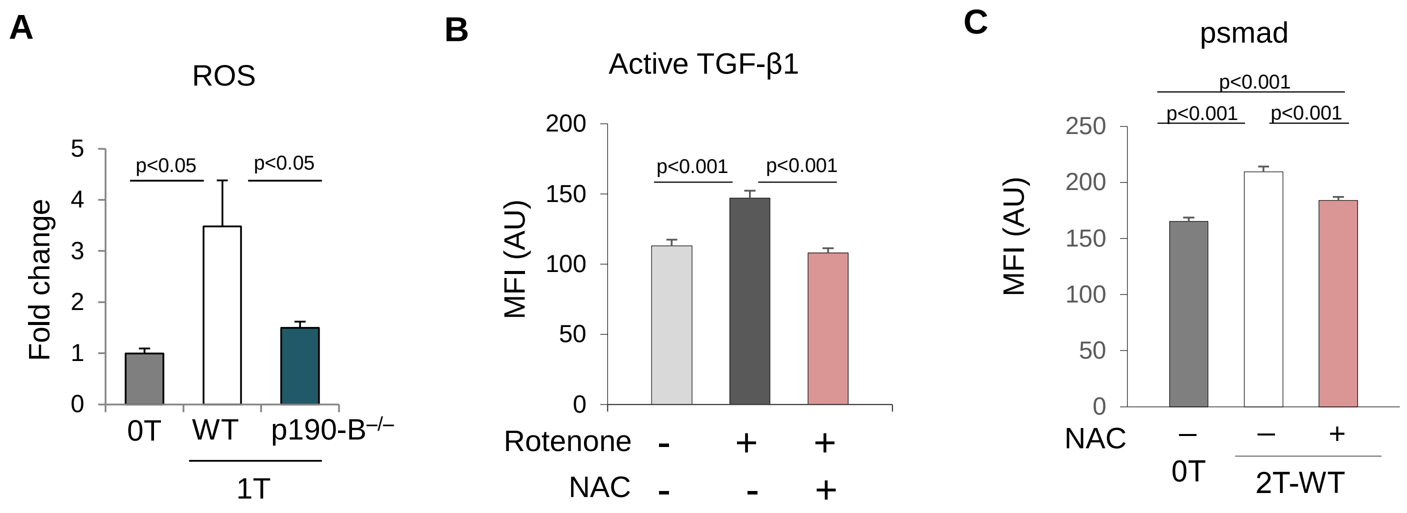

Supplementary figure 9 Increase in reactive oxygen species levels changes HSPC differentiation via TGF-β1 signaling. (A) ROS detection by DCFDA staining by flow cytometric analysis in 0T and 1T WT and p190-B-/- LSK-SLAM cells (n=6 independent samples, 2 independent experiments). **(B)** Detection of aTGF-β by immuno-fluorescence staining: 0T LSK-SLAM cells were treated with rotenone alone (1µM) and rotenone+ NAC (100 µM) for 24 hours and immuno-stained for aTGF-β levels in 0T-LSK-SLAM cells. (n=3 samples, minimum 40-50 cells per experiment were analyzed in each groups). **(C)** 2T WT mice were treated with NAC in drinking water (10mg/ml) for 5-8 weeks post-transplant. 2T-WT mice not treated with NAC ant 0T mice were used as controls. Immuno-fluorescence staining for psmad2 in LSK-SLAM cells (n=2 independent experiments, 40-60 cells per group, mean ± SEM). P value was calculated using 2 tailed un-paired t test between the groups as indicated.

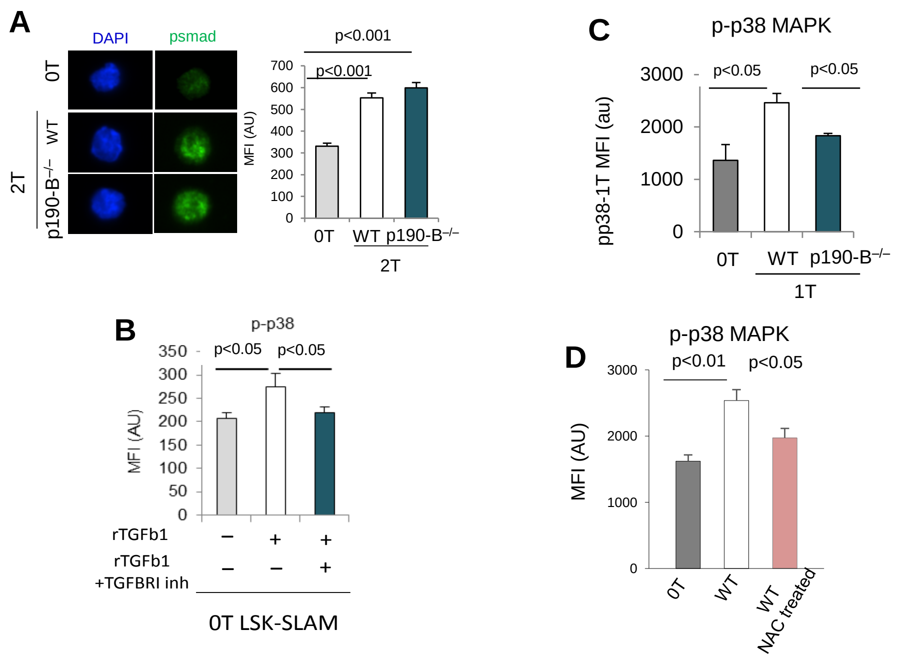

Supplementary figure 10. p38MAPK mediates TGF-β effect on HSPC functions. (A) Immuno-staining of psmad2 in 0T, 2T WT and p190-B-/- LSK-SLAM cells, four months following transplant. Left panel are representative images, scale bar 10μm. Bar graph is mean fluorescence intensity in each group (mean ± SEM; 35- 50 cells from 2 independent experiments). **(B)** 0T LSK-SLAM were treated in vitro with rTGF-β1 (10pg/ml) in the presence or absence of TGFBRI-Inhibitor and pp38 was evaluated by immunofluorescence staining (mean ± SEM, n=50 cells from 2 independent experiments). **(C)** p-p38MAPK detection by flow cytometry in 0T and 1T WT and p190-B-/- LSK-SLAM cells (mean ± SEM, n=5-6 from 2 independent experiments). **(D)** 2T-WT mice were treated with NAC in drinking water (10mg/ml) for 5-8 weeks post-transplant. 2T-WT mice not treated with NAC ant 0T mice were used as controls. Detection of p-p38MAPK by flow cytometry in LSK-SLAM cells is shown. (n=6 independent samples, 2 independent experiments). P value were calculated using 2 tailed un-paired t test between the groups as indicated.

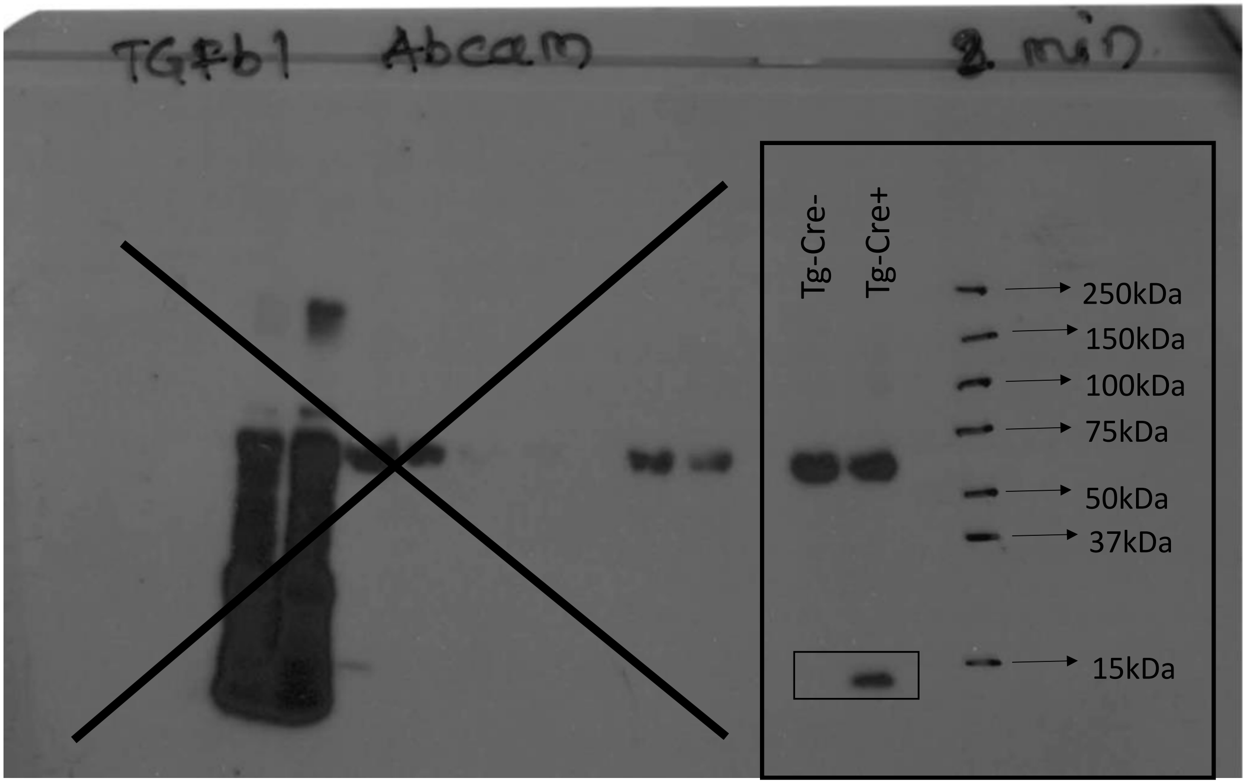

Supplementary Figure 11. Uncropped western blot for figure 6b

Supplementary Table 1. Paired daughter cell assay

	Control	WT	P190-B-/-
Symmetric Division	39	19	24
Asymmetric Division	3	18	2
Committed	4	0	3

In vitro paired daughter cell assay of 2T WT, p190-B-/- and 0T LSK-SLAM cells as shown in figure 1F. Table shows actual numbers of single cells that gave rise to symmetric, asymmetric or committed cell divisions in each group. N= 35 to 55 pairs obtained from 3 or more independent experiments

Supplementary Table 2. Effect of TGFBRI-Inh2 on peripheral blood

	**WT-DMSO injected**	**WT – TGFBRI-inh2 injected**
**WBC**	3.65 ± 0.33	4.02 ± 0.39
RBC	9.01 ± 0.22	9.03 ± 0.51
Hemoglobin	13.08 ± 0.37	13.14 ± 0.86
Hematocrit	46.03 ± 0.95	46.38 ± 2.75
Platelet	545.2 ± 34.6	610.7 ± 42.32

Secondary recipient mice transplanted using WT cells were treated in vivo with DMSO or TGF-βRI kinase inhibitor II [TGFBRI-Inh2] for four weeks (data from 2-3 independent experiments). PB and counts of mice as indicated (13 – 15 mice per group).

Supplementary Table 3. Effect of rTGF-β1 and p38 inhibitor on p190-B$^{-/-}$ fetal liver LSK-SLAM engraftment

	Chimera > 1% (+ve mice)	Chimera < 1% (-ve mice)	Total mice transplanted	p value
**p190-B $^{-/-}$**	8	4	12	
p190-B$^{-/-}$ + rTGFβ1	2	10	12	<0.0001#
p190-B$^{-/-}$ + rTGFβ1 + p38 inhi	4	6	10	0.0005$

200 LSK-SLAM cells from p190-B$^{-/-}$ fetal liver were treated with either rTGF-β1 alone or rTGF-β1 + SB203580 for 48h; mixed with 200,000 CD45.1+ cells and were transplanted in recipient mice. Table shows number of mice showing >1% and < 1% chimera within myeloid population post 16 weeks of transplant. (2-3 independent experiments). P value was calculated by Fisher exact test; p190-B$^{-/-}$ vs. p190-B$^{-/-}$ + rTGF-β1(<0.0001 in FL) and p190-B$^{-/-}$ + rTGF-β1vs.p190-B$^{-/-}$ + rTGF-β1+ p38 inhi (0.0005 in FL). P value by chi square test was < 0.0001 in both sets of transplant.
